# Reciprocal Nucleopeptides as the Ancestral Darwinian Self-Replicator

**DOI:** 10.1093/molbev/msx292

**Published:** 2017-11-08

**Authors:** Eleanor F Banwell, Bernard M A G Piette, Anne Taormina, Jonathan G Heddle

**Affiliations:** 1 Heddle Initiative Research Unit, RIKEN, 2-1 Hirosawa, Wako, Saitama 351-0198, Japan; 2 Department for Mathematical Sciences, Durham University, Durham, United Kingdom; 3 Bionanoscience and Biochemistry Laboratory, Malopolska Centre of Biotechnology, Jagiellonian University, Krakow, Poland

**Keywords:** Initial Darwinian Ancestor, abiogenesis, RNA world, protein world, nucleopeptide replicator, reciprocal replicator, polymerase, ribosome, evolution, early earth, hypercycle

## Abstract

Even the simplest organisms are too complex to have spontaneously arisen fully formed, yet precursors to first life must have emerged ab initio from their environment. A watershed event was the appearance of the first entity capable of evolution: the Initial Darwinian Ancestor. Here, we suggest that nucleopeptide reciprocal replicators could have carried out this important role and contend that this is the simplest way to explain extant replication systems in a mathematically consistent way. We propose short nucleic acid templates on which amino-acylated adapters assembled. Spatial localization drives peptide ligation from activated precursors to generate phosphodiester-bond-catalytic peptides. Comprising autocatalytic protein and nucleic acid sequences, this dynamical system links and unifies several previous hypotheses and provides a plausible model for the emergence of DNA and the operational code.

## Introduction

In contrast to our good understanding of more recent evolution, we still lack a coherent and robust theory that adequately explains the initial appearance of life on Earth (abiogenesis). In order to be complete, an abiogenic theory must describe a path from simple molecules to the Last Universal Common Ancestor (LUCA), requiring only a gradual increase in complexity.

The watershed event in abiogenesis was the emergence of the Initial Darwinian Ancestor (IDA): the first self-replicator (ignoring dead ends) and ancestral to all life on Earth (Yarus 2011). Following the insights of von Neumann, who proposed the kinematic model of self-replication (Kemeny 1955), necessary features of such a replicator are: Storage of the information for how to build a replicator; a processor to interpret information and select parts; an instance of the replicator.

In order to be viable, any proposal for the IDA’s structure must fit with spontaneous emergence from prebiotic geochemistry and principles of self-replication. Currently, the most dominant abiogenesis theory is the “RNA world,” which posits that the IDA was a self-replicating ribozyme, that is, an RNA-dependent RNA polymerase (Cech 2012). Although popular, this theory has problems (Kurland 2010). For example, while it is plausible that molecules with the necessary replication characteristics can exist, length requirements seem to make their spontaneous emergence from the primordial milieu unlikely, nor does the RNA world explain the appearance of the operational code (Noller 2012; Robertson and Joyce 2012). Furthermore, it invokes three exchanges of function between RNA and other molecules to explain the coupling of polynucleotide and protein biosynthesis, namely transfer of information storage capability to DNA and polymerase activity to protein as well as gain of peptide synthesis ability. This presents a situation in which no extant molecule continues in the role it initially held. Others have posited peptide and nucleopeptide worlds as solutions.

The peptide world theory proposes a spontaneously occurring self-replicating peptide with RNA synthesis, DNA and the operational code appearing later, and possible self-replicating mechanisms of peptides have been explored (Fox and Harada 1958; Lee et al. 1996). Nucleopeptide theories require that the replicator consist of both peptides and nucleic acids and may involve their covalent linkage or (as in our proposal) noncovalent conjugation. Covalently linked nucleopeptides include nucleobase-containing peptides such as PNA which has been mooted as a possible precursor to the RNA world (Miller 1997) and possible RNA-interacting nucleo-∈-peptides have been synthesized (Roviello et al. 2009; Nelson et al. 2000). Both the peptide world and nucleopeptide theories consist of single molecular classes and therefore suffer the same exchange of function problems as the RNA-world theory. To the best of our knowledge, no single theory has emerged that parsimoniously answers the biggest questions.

Here, we build on several foregoing concepts to propose an alternative theory based around a nucleopeptide reciprocal replicator that uses its polynucleotide and peptide components according to their strengths, thus avoiding the need to explain later exchange of function and coupling. We advocate a view of the IDA resulting from a biochemical system which we describe as a dynamical system, that is, a system of equations describing the changes that occur over time in the self-replicator presented here, and we demonstrate that such an entity is both mathematically consistent and complies with all the logical requirements for life. While necessarily wide in view we hope that this work will provide a useful framework for further investigation of this fundamental question.

## Model and Results

### Solving the Chicken and Egg Problem

**Fig. 1.**
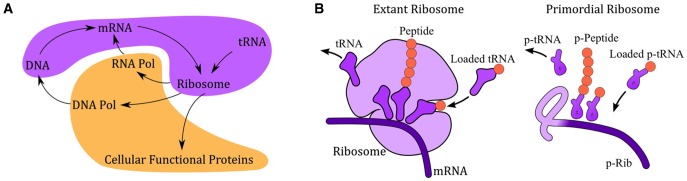
Replication schemes. (*a*) This simplified cellular replication schematic is common to all life today and likely reflects the ancestral form present in LUCA. Shading by molecule type (purple for nucleic acid and orange for protein), reveals a reciprocal nucleopeptide replicator. Although the ribosome is a large nucleoprotein complex, the catalytic centre has been shown to be a ribozyme (Moore and Steitz 2003) and so it is shaded purple in this scheme. (*b*) Comparison of the method of action of the extant ribosome with the proposed primordial analogue (components are shaded like for like). Today, tRNA molecules (mid purple) loaded with amino acids (orange) bind the mRNA (dark purple) in the ribosome (light purple), which co-ordinates and catalyses the peptidyl-transferase reaction. Although the present day modus operandi is regulated via far more complex interactions than the primordial version, the two schemes are fundamentally similar. Mixed nucleic acid structures, one performing a dual function as primordial mRNA and primordial ribosome (p-Rib) and a second functioning as a primordial tRNA (p-tRNA), provide a system wherein the former structure templates amino acid-loaded molecules of the latter.

Given that any IDA must have been able to replicate in order to evolve, extant cellular replication machinery is an obvious source of clues to its identity. Common ancestry means that features shared by all life were part of LUCA. By examining the common replication components present in LUCA, and then extrapolating further back to their simplest form, it is possible to reach a pre-LUCA, irreducibly complex, core replicator (fig. 1).

We see that in all cells, the required functions of a replicator are not carried out by a single molecule or even a single class of molecules, rather they are performed variously by nucleic acids (DNA, RNA) and proteins. When viewed by molecular class, the replicator has two components and is reciprocal in nature: polynucleotides rely on proteins for their polymerization and vice versa. The question of which arose first is a chicken and egg conundrum that has dogged the field since the replication mechanisms were first elucidated (Giri and Jain 2012). In this work, we suggest that, consistent with common ancestry and in contrast with the RNA world theory, the earliest replicator was a two—rather than a one—component system, composed of peptide and nucleic acids.

### Assumptions of the Model

We postulate that, in a nucleopeptide reciprocal replicator, the use of each component according to its strengths could deliver a viable IDA more compatible with evolution to LUCA replication machinery. Although seemingly more complex than an individual replicating molecule, the resulting unified abiogenesis theory answers many hard questions and is ultimately more parsimonious. The model does not consider in detail the chemistry of how the building blocks that constitute the IDA (short peptides and nucleic acids) came about as these details are covered in the cited literature (see for example, Saladino et al. 2012; Patel et al. 2015; Da Silva et al. 2015; Leman et al. 2004; Liu et al. 2014; Martin et al. 2008). Rather, we concentrate on the important question of the mathematical validity of the IDA in terms of its ability to sustainably self-replicate, without which it would not be a valid system. In constructing our model, we make the following assumptions:

*(i) The existence of random sequences of short strands of mixed nucleic acids (XNA) likely consisting of ribonucleotides, deoxyribonucleotides and possibly other building blocks able topolymerizewith nucleotide chains, as well as the existence of random amino acids and short peptides produced abiotically.*

For this first assumption we have supposed a pool of interacting amino acids, nucleotides and related small molecules as well as a supply of metal ions, other inorganic catalysts and energy. The precise understanding of the “metabolic” reactions in which these precursor building blocks were formed is in itself an extremely important question but is not considered here as a number of potential early earth conditions and reaction pathways resulting in these outcomes have already been proposed, including the formamide reaction (Saladino et al. 2012) and cyanosulfidic chemistries (Patel et al. 2015). Recent experimental models of alkaline hydrothermal vents have even succeeded in producing various organic molecules including ribose and deoxyribose (Herschy et al. 2014). Pools of pure molecules are unlikely; instead, mixtures would likely have comprised standard and nonstandard amino acids as well as XNAs with mixed backbone architectures, being, in their simplest forms, mixtures of deoxy- and ribonucleotides (Trevino et al. 2011; Pinheiro et al. 2012) with other building blocks being possible. For simplicity we sometimes refer to XNAs as “polynucleotides.” Such conditions would be conducive to the occasional spontaneous covalent attachment of nucleotides to each other to form longer polymer chains (Da Silva et al. 2015).

(ii) The existence of abiotically aminoacylated short XNA strands (primordial tRNAs (p-tRNAs))

The second assumption is potentially troubling as amino acid activation is slow and thermodynamically unfavorable. However, amino acylation has been investigated in some detail and has been shown to be possible abiotically including, in some cases, the abiotic production of activated amino acids (Illangasekare et al. 1995; Leman et al. 2004; Giel-Pietraszuk and Barciszewski 2006; Lehmann et al. 2007; Turk et al. 2010; Liu et al. 2014). A pool of activated amino acids allows us to presume a fast rate of charging of p-tRNAs meaning that we can assume that the rate of charged p-tRNA formation is proportional to the concentration of free amino acids. Taken together these data suggest that multiple small amino-acylated tRNA-like primordial XNAs could have arisen. Though likely being XNA in nature, we refer to them as p-tRNA, reflecting their function. A similar nomenclature applies to p-Rib and p-mRNA.

*(iii) Conditions that allow a codon/anticodon interaction between two or more charged p-tRNA for sufficient time and appropriate geometry to allow peptide bond formation*, *that is*, *the functionality of a primordial ribosome (p-Rib)*

**Fig. 2.**
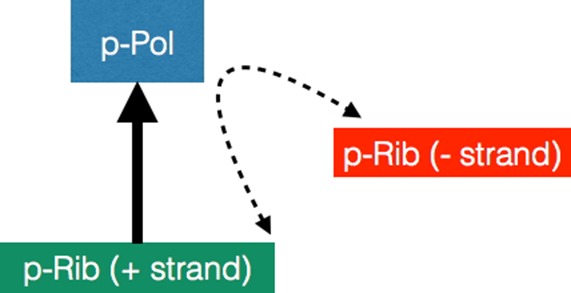
Models of primitive polymerization reactions. An XNA strand can function like a primordial ribosome (p-Rib) whereby one strand (+ strand) can template the production of a primordial polymerase (p-Pol) as indicated by the solid arrow. The action of this p-Pol is represented by the double-headed dotted arrow whereby it acts on the p-Rib (+ strand) to catalyze synthesis of the complementary sequence (− strand) and also on the − strand to produce more of the + strand.

Our proposed p-Rib is an extreme simplification of the functionality of both the present day ribosome and mRNA (fig. 1). Initially, the p-Rib need only have been a (close to) linear assembly template for the p-tRNAs to facilitate the peptidyl transferase reaction through an increase in local concentration. This mechanism is simple enough to emerge spontaneously and matches exactly the fundamental action of the extant ribosome (fig. 2). The idea that a p-Rib may have an internal template rather than separate mRNA molecules and that an RNA strand could act as a way to bring charged tRNAs together has previously been suggested (Schimmel and Henderson 1994; Wolf and Koonin 2007; Morgens 2013) and is known as an “entropy trap” (Sievers et al. 2004; Ruiz-Mirazo et al. 2014). The concept has been demonstrated to be experimentally viable (Tamura and Schimmel 2003) although in the latter case it is the primordial ribosomal rRNA strand itself that provides one of the two reacting amino acids.

A functional operational system requires preferential charging of particular p-tRNAs to specific amino acids. Although there is evidence for such relationships in the stereochemical theory (Woese 1965; Yarus et al. 2009), so far unequivocal proof has been elusive (Yarus et al. 2005; Koonin and Novozhilov 2009). However, there is sufficient evidence to suggest at least a separation along grounds of hydrophobicity and charge using just a two-base codon (Knight and Landweber 2000; Biro et al. 2003; Rodin et al. 2011). Furthermore only a reduced set of amino acids (Angyan et al. 2014)—possibly as few as four (Ikehara 2002)—need to have been provided in this way. The “statistical protein” hypothesis proposes that such a weak separation may have been sufficient to produce populations of active peptides (Ikehara 2005; Vetsigian et al. 2006). Such “primordial polymerases” (p-Pol) need only have been small (see below) and spontaneous emergence of a template coding loosely for such a sequence seems plausible. The failure rate of such syntheses would be high but a p-Rib using the outlined primordial operational code to produce statistical p-Pol peptides could have been accurate enough to ensure its own survival.

*(iv) Theviability of averyshortpeptidesequence tofunction as an RNA-dependent RNApolymerase*

Templated ligation is often proposed as a primordial self-replication mechanism, particularly for primitive replication of nucleic acid in RNA world type scenarios. However, these are associated with a number of problems as mentioned earlier. In addition, extant RNA/DNA synthesis proceeds via terminal elongation (Paul and Joyce 2004; Vidonne and Philp 2009). To be consistent with the mechanism present in LUCA and pre-LUCA, the p-Pol should, preferably, have used a similar process.

During templated ligation, a parent molecule binds and ligates short substrates that must then dissociate to allow further access, but the product has greater binding affinity than the substrates and dissociation is slow. This product inhibition results in parabolic growth and limits the usefulness of templated ligation for replication (Issac and Chmielewski 2002). Conversely, in 1D sliding (or more accurately jumping), the catalyst may dock anywhere along a linear substrate and then diffuse by “hops” randomly in either direction until it reaches the reaction site; a successful ligation reaction has little impact on binding affinity and leaves the catalyst proximal to the next site. For simplicity our model assumes a single binding event between p-Pol and p-Rib followed by multiple polymerization events. A p-Pol proceeding via 1D sliding could catalyze phosphodiester bond formation between nucleotides bound by Watson and Crick base-pairing to a complementary XNA strand. Because p-Pol activity would be independent of substrate length, a relatively small catalyst could have acted on XNAs of considerable size. From inspection of present day polymerases such a peptide may have included sequences such as DxDGD and/or GDD known to be conserved in their active sites and consisting of the amino acids thought to be amongst the very earliest in life (Iyer et al. 2003; Koonin 1991).

In our simple system any such p-Pol must be very short to have any realistic chance of being produced by the primitive components described. We must therefore ask if there is evidence that small (e.g. <11 amino acid) peptides can have such a catalytic activity. Catalytic activity in general has been demonstrated for molecules as small as dipeptides (Kochavi et al. 1997). For polymerase activity in particular, it is known that randomly produced tripeptides can bind tightly and specifically to nucleotides (Schneider et al. 2000; McCleskey et al. 2003). We suggest that a small peptide could arise with the ability to bind divalent metal ions, p-Rib and incoming nucleotides. It is interesting to note that small peptides can assemble into large and complex structures (Bromley et al. 2008; Fletcher et al. 2013) with potentially sophisticated functionality: di- and tripeptides can self-assemble into larger nanotubes and intriguingly it has even been suggested that these structures could have acted as primitive RNA polymerases (Carny and Gazit 2005).

In summary, the essence of the model is that on geological timescales, short linear polynucleotides may have been sufficient to template similar base-pairing interactions to those seen in the modern ribosome with small amino-acylated adapters. Given that the majority of ribosome activity stems from accurate substrate positioning, such templating could be sufficient to catalyze peptide bond formation and to deliver phosphodiester-bond-catalytic peptides. As backbone ligation reactions are unrelated to polynucleotide sequence, these generated primordial enzymes could have acted on a large subset of the available nucleic acid substrates, in turn producing more polynucleotide templates and resulting in an autocatalytic system.

## Mathematical Model

The IDA described above is attractive both for its simplicity and continuity with the existing mixed (protein/nucleic acid) replicator system in extant cells. However, the question remains as to whether such a system is mathematically consistent, could avoid collapse and instead become self-sustaining. The number of parameters and variables needed to analyze the system in its full complexity is such that one is led to consider simplified models which nevertheless capture essential features of interest. Here we consider a simple model of RNA–protein self-replication.

### Constituents

The main constituents of the simplest model of XNA-protein self-replication considered here (see also figs. 1*b* and 2) are a pool of free nucleotides and amino acids, polypeptide chains—including a family of polymerases—and polynucleotide chains as well as p-tRNAs loaded with single amino acids.

We introduce some notations. Generically, we consider polymer chains Π made of *n types* of building blocks labeled 1,…,n. In our models, the polymer chains are polypeptides and polynucleotides, and the building blocks are amino acids and codons respectively. With a slight abuse of language, we call the number of constituents (building blocks) of a polymer chain its *length*. So hereafter, “lengths” are dimensionless. The order in which these constituents appear in any chain is biologically significant, and we encode this information in finite ordered sequences of arbitrary length *L* denoted $S\{L\}=(s_{1},s_{2},\ldots ,s_{L})$, whose elements $s_{j},\;j=1,\ldots L$ label the building blocks forming the chains, in the order indicated in the sequences. Each element *s_j_* in the sequence S{L} is an integer in the set {1,…,n} which refers to the type of building block occupying position *j* in the chain. There are therefore *n^L^* sequences of length *L* if the model allows *n* types of building blocks. For instance, the sequence S{5}=(1,4,3,1,3) in a model with, say, *n* = 4 types of building blocks (amino acids or codons), corresponds to a polymer chain of length 5 whose first component is a type 1 building block, the second component is a type 4 and so on. Given a sequence S{L}, we introduce subsequences $S\{L,\;j\}=(s_{1},s_{2},\ldots s_{j})$ (resp. $\hat{S\{L,\;j\}}=(s_{L-j+1},s_{L-j+2},\ldots s_{L})$), j=1,…L, whose elements are the *j* leftmost (resp. rightmost) elements of S{L}. In particular, $S\{L,\;L\}\equiv \hat{S\{L,\;L\}}\equiv S\{L\},\;S\{L,\;1\}=s_{1}$ and $\hat{S\{L,1\}}=s_{L}$. We write

$$S\{L\}=(S\{L,\;L-\ell \},\hat{S\{L,\;\ell \}}),\;0<\ell <L.$$

In what follows we sometimes refer to families of polymer chains differing only by their length and obtained by removing some rightmost building blocks from a chain of maximum length $L_{\text{max}}$. Denoting by $\Pi_{\ell }^{S}$ a polymer chain of length *ℓ* and sequence S{ℓ} or subsequence S{L,ℓ}, both having *ℓ* elements with L>ℓ, the family of polymer chains obtained from a chain of maximal length $L_{\text{max}}$ and sequence $S\{L_{\text{max}}\}$ is given by ${\left\{\Pi_{\ell }^{S}\right\}}_{\ell =1,2,\ldots L_{\text{max}}}$.

**Fig. 3.**
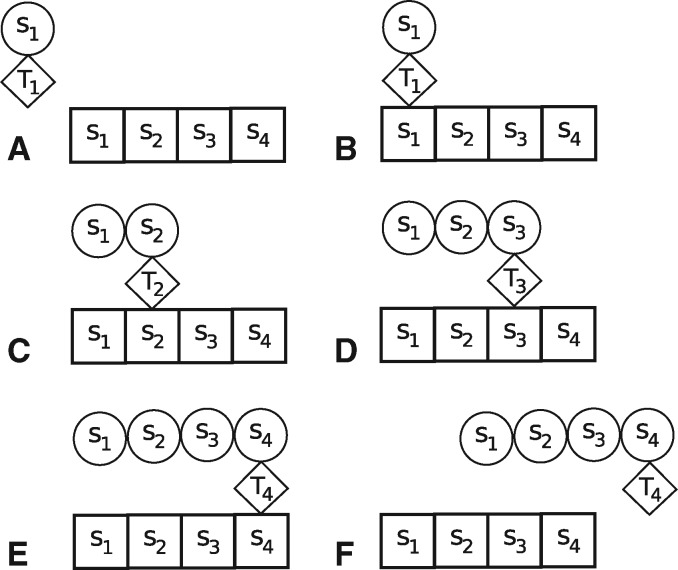
Mechanism (B): Polypeptide polymerization in our model. The square boxes represent the codons of a polynucleotide chain (here, of length *L* = 4) and the circles represent amino acids. The p-tRNA molecules are labeled $T_{1},\ldots ,T_{4}$.

In the specific case of XNA/polynucleotide chains entering our model, we use Π=R and the sequences are generically labeled as α{ℓ}. Their elements correspond to types of codons, and the complementary codon sequences in the sense of nucleic acids complementarity are $\bar{\alpha }\{\ell \}$. Therefore, a large class of XNA strands of length *ℓ* and sequence α{ℓ} are denoted by $R_{\ell }^{\alpha }$, and in particular, $R_{1}^{\alpha_{1}}$ is a codon of type *α*_1_. Besides the generic sequences α{ℓ} introduced above, a sequence denoted $\pi \{L_{\text{max}}\}$, together with its subsequences $\pi \{L_{\text{max}},\;\ell \}$ and $\hat{\pi \{L_{\text{max}},\;\ell \}}$ for $\ell =1,\ldots L_{\text{max}}$ play a specific role: they correspond to polynucleotide chains that template the polymerization of a family of primordial peptide polymerases (p-Pol) through a process described in the next subsection, see also figure 3. Using Π=P to denote polypeptide chains, this family of polymerases derived from $P_{L_{\text{max}}}$ of maximal length $L_{\text{max}}$, is ${\left\{P_{\ell }^{\pi }\right\}}_{\ell =2,\ldots ,L_{\text{max}}}$. These polymerases are such that $P_{\ell }^{\pi }=P_{\ell -1}^{\pi }+P_{1}^{\pi_{\ell }}$, with $P_{1}^{\pi_{\ell }}$ an amino acid $\pi_{\ell }$. We use the notation $P^{\pi }$ for a generic polymerase in the family. Alongside these polymerases, generic polypeptide chains of length *ℓ* and sequence α{ℓ} are labeled as $P_{\ell }^{\alpha }$. Proteins of length 1, $P_{1}^{\alpha_{1}}$, are single amino acids of type $\alpha_{1}$.

### RNA–Protein Replication Scenario

**Fig. 4.**
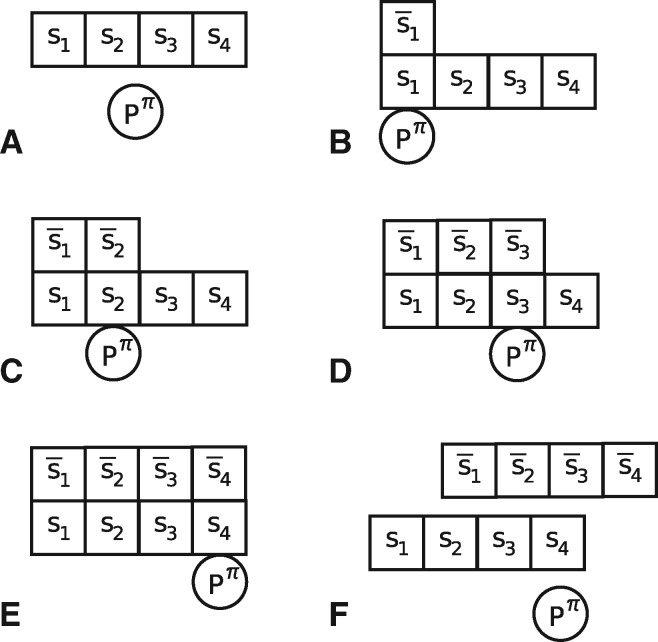
First phase of Mechanism (C): Polymerization of the complementary polynucleotide chain $R_{L}^{\bar{S}}$ catalyzed by a primordial polymerase $P^{\pi }$.

The scenario relies on three types of mechanisms:

- The *spontaneous* polymerization of polynucleotide and polypeptide chains, assumed to occur at a very slow rate, and their depolymerization through being cleaved in two anywhere along the chains at a rate independent of where the cut occurs.
- The *nonspontaneous* polypeptide polymerization occurring through a polynucleotide chain $R_{L}^{S}$ on which several p-tRNA molecules loaded with an amino acid dock and progressively build the polypeptide chain. More precisely, each codon of type *s* of the polynucleotide chain binds with a p-tRNA, itself linked to an amino acid of type *s*. Note that we assume the same number *n* of types of codons and amino acids. This leads to a chain of amino acids matching the codon sequence S{L} of the polynucleotide chain. The process is illustrated in figure 3 for a polypeptide chain of length *L* = 4 and amino acid sequence $S\{4\}=(s_{1},s_{2},s_{3},s_{4})$.
- The duplication of a polynucleotide chain $R_{L}^{S}$, of length $L\geq \ell_{\pi \text{min}}$, as a two-step process. In the first step, a polypeptide polymerase $P^{\pi }$, obtained by polymerization via mechanism (B) using a polynucleotide $R_{L}^{\pi }$, scans the polynucleotide chain $R_{L}^{S}$ to generate its complementary polynucleotide chain $R_{L}^{\bar{S}}$. This is shown in figure 4. The resulting polynucleotide chain $R_{L}^{\bar{S}}$ is then used to generate a copy of the original polynucleotide chain $R_{L}^{S}$ via the same mechanism (C).

The replicator crudely operates as follows:

- Mechanism (A) provides a small pool of polymer chains; among them, one finds short strands of XNA with dual function (p-mRNA and p-Rib)
- Mechanism (B) provides polypeptide chains, including the polymerases (p-Pol, called $P^{\pi }$ here), by using the XNA produced through Mechanism (A) and Mechanism (C)
- $P^{\pi }$ are involved, through Mechanism (C), in the duplication of polynucleotides present in the environment, including the strands of XNA that participate in the very production of $P^{\pi }$

### Reactions Driving the Replication and Physical Parameters

For simplicity, we consider the polymerization of polypeptide chains and the duplication of polynucleotide chains as single reactions where the reaction rates take into account all subprocesses as well as failure rates.

This leads to the following schematic reactions:

Mechanism  (A)

(1) $$R_{L}^{S}+R_{1}^{s_{L+1}}\to R_{L+1}^{S},$$

(2) $$R_{L}^{S}\to R_{L_{-}\ell }^{S}+R_{\ell }^{\hat{S}}\;\ell =1,\ldots ,L-1,$$

(3) $$P_{L}^{S}+P_{1}^{s_{L+1}}\to P_{L+1}^{S},$$

(4) $$P_{L}^{S}\to P_{L_{-}\ell }^{S}+P_{\ell }^{\hat{S}}\;\ell =1,\ldots ,L-1.$$

Mechanism  (B)

(5) $$R_{L}^{S}+L\times TRP\to R_{L}^{S}+P_{L}^{S}.$$

Mechanism  (C)

(6) $$R_{L}^{S}+L\times R_{1}\overset{P^{\pi }}{\to }R_{L}^{S}+R_{L}^{\bar{S}},$$

where *TRP* denotes p-tRNA loaded with a single amino acid.

The parameters for these reactions are (see the Supplementary Material online for more details on the estimation of the parameter values):

- $K_{R}^{+}$: polymerization rate of polynucleotide chains (eq. 1); we have estimated the catalyzed XNA polymerization rate to be $4.2\times 10^{-7}\;{\text{mol}}^{-1}\;m^{3}\;s^{-1}$.
- $K_{R}^{-}$: depolymerization rate of polynucleotide chains (hydrolysis) (eq. 2); taken to be $8\times 10^{-9}s^{-1}$.
- $K_{P}^{+}$: polymerization rate of polypeptide chains (eq. 3); we have estimated it to be $2.8\times 10^{-21}\;{\text{mol}}^{-1}\;m^{3}\;s^{-1}$.
- $K_{P,S,L}^{-}$: depolymerization rate of polypeptide chains of length *L* and sequence *S* (eq. 4); we have estimated it to be in the range $4\times 10^{-11}\;s^{-1}-5.1\times 10^{-6}\;s^{-1}$.
- $k_{P,\;L}^{+}$: polymerization rate of a polypeptide of length *L* from the corresponding polynucleotide chain (eq. 5). It is reasonable to assume that $k_{P,L}^{+}=k_{P,1}^{+}/L$ and we have estimated $k_{P,\;1}^{+}$ to be $0.1\;{\text{mol}}^{-1}\;m^{3}\;s^{-1}$.
- *Z*: the rate at which a polymerase attaches to a polynucleotide chain (eq. 6) which we have estimated to be $10^{6}\;{\text{mol}}^{-1}\;m^{3}\;s^{-1}$.
- *h_R_*: the rate of attachment of a free polynucleotide to a polynucleotide chain attached to a p-Pol (eq. 6). We have estimated it to be $10^{6}\;{\text{mol}}^{-1}\;m^{3}\;s^{-1}$.
- $k_{\text{step}}$: the rate at which a polymerase moves by one step on the polynucleotide (eq. 6). We have estimated it to be in the range $2\times 10^{-2}\;s^{-1}$–$4\times 10^{-5}\;s^{-1}$.

We now argue that the three parameters $Z,h_{R}$ and $k_{\text{step}}$ enter the dynamical system for the polymer concentrations in our model as two *physical* combinations denoted K(L) and $P_{b}$ that we describe below.

First recall that we assume the existence of a pool of nucleotides, amino acids and p-tRNA. The amount of *free* nucleotides and amino acids is taken to be the difference between the total amount of these molecules and the total amount of the corresponding polymerized material, ensuring total conservation.

We denote the concentration of polypeptide and polynucleotide chains respectively by $P_{L}^{\alpha },P_{L}^{\pi },P_{L}^{\bar{\pi }}$ and $R_{L}^{\alpha },R_{L}^{\pi },R_{L}^{\bar{\pi }}$, all expressed in $\text{mol}\;m^{-3}$$molm^{-3}$. In particular, *P*_1_ and *R*_1_ are the concentrations of each type of free amino acids and nucleotides respectively, and we assume, for simplicity, that all types of amino acids/codons are equally available.

We also assume that the amount of loaded p-tRNA, $C_{p-\text{tRNA}}$, remains proportional to the amount of free amino acids and that the concentration of p-tRNA is larger than *P*_1_ so that most amino acids are loaded on a p-tRNA. With these conventions, one has

(7) $$C_{p-\text{tRNA}}=k_{t}P_{1}\text{ with }k_{t}\approx 1.$$

#### Total Reaction Rate K(L) of Polynucleotide Polymerization

If a complex reaction is the result of one event at rate *K*, and *m* other, identical, events at rate *k*, the average time to complete the reaction is the sum of the average times for each event. Hence the reaction rate is given by

(8) $$\tilde{K}(K,k,m)={\left(\frac{1}{K}+\frac{m}{k}\right)}^{-1}=\frac{Kk}{mK+k}.$$

One such complex reaction in our model is the polymerization of a polynucleotide chain of length *L*, say, from its complementary chain (second phase of Mechanism (C)). Polymerases are characterized by the polymerizing efficiency which, we assume, increases with *ℓ*, up to $L_{\text{max}}$. The first step in polymerization requires a polymerase to attach itself to the template polynucleotide. This is only possible if the template polynucleotide has a minimum length, which we assume to be $\ell_{\pi \text{min}}$. In the following, we assume that polymerases can polymerize polynucleotide chains of any length greater or equal to $\ell_{\pi \text{min}}$. The corresponding reaction rate is given by $Z\;P_{\ell }^{\pi }$ for a polymerase of length $\ell \geq \ell_{\pi \text{min}}$.

The free nucleotides must then attach themselves to the polynucleotide–polymerase complex and the polymerase must move one step along the polynucleotide. The rate for each of these *L* steps is

(9) $$k_{R+}=\frac{k_{\text{step}}\;h_{R}R_{1}}{k_{\text{step}}+h_{R}R_{1}},$$

and hence, the rate of polymerization for a polynucleotide of length *L* and polymerase of length *ℓ* is $\tilde{K}(Z\;P_{\ell }^{\pi },\;k_{R+},\;L)$. However, it is assumed that polymerases of several lengths are available and therefore, the total rate is given by

(10) $$K(L)=\{\begin{array}{cc} \sum_{\ell =\ell_{\pi \text{min}}}^{L_{\text{max}}}\tilde{K}(ZP_{\ell }^{\pi },\;k_{R+},\;L)\;W_{\ell }, & L\geq \ell_{\pi \text{min}}\\ 0 & L<\ell_{\pi \text{min}}, \end{array}$$

where it is understood that $\ell_{\pi \text{min}}$ is the lower bound length for polymerase activity and $W_{\ell }$ is a quality factor given by

(11) $$W_{\ell }=\{\begin{array}{cc} \frac{\ell -\ell_{\pi \text{min}}+1}{\ell_{\pi \text{max}}-\ell_{\pi \text{min}}+1}\; & \ell_{\pi \text{min}}\leq \ell \leq \ell_{\pi \text{max}}\\ 1 & \ell_{\pi \text{max}}<\ell \leq L_{\text{max}}. \end{array}$$

Indeed, we expect long polymerases to be more efficient, so $W_{\ell }$ is taken to increase with *ℓ* in the range $\ell_{\pi \text{min}}\leq \ell \leq \ell_{\pi \text{max}}$, while polymerases of length $\ell >\ell_{\pi \text{max}}$ have the same level of activity as those with length $\ell =\ell_{\pi \text{max}}$, that is, $W_{\ell \;>\;\ell_{\text{max}}}=1$.

To avoid proliferation of parameters in our simulations, we have taken $\ell_{\pi \text{max}}=L_{\text{max}}$, where $L_{\text{max}}$ is the maximal polynucleotide chain’s length.

#### Binding Probability $P_{b}$ of a Polynucleotide and a Polymerase of Length *L*

First note that it takes *L* times longer to synthesize a polypeptide chain of length *L* from its corresponding polynucleotide chain than it takes for one amino acid to bind itself to the polynucleotide. The rate is thus given by $k_{P,\;L}^{+}\;P_{1}=(k_{P,\;1}^{+}/L)\;P_{1}$.

We now offer some considerations on depolymerization. We assume that if a polymer $\Pi_{L}^{S}$ depolymerizes, it does so by (potentially consecutive) cleavings. In the first step, $\Pi_{L}^{S}$ can cleave in *L* – 1 different positions, resulting in two smaller chains *L*_1_, *L*_2_ with $L=L_{1}+L_{2}$ and $1\leq L_{1,2}\leq L-1$. This is the origin of the factor (L−1) in the terms describing the depolymerization of polymer chains in the dynamical systems equations presented in the next subsection.

The concentration variations resulting from such depolymerizations must be carefully evaluated. A polymer $\Pi_{L}^{S}$ of length *L* and sequence *S*, where *S* stands for any of *α*, *π* or $\bar{\pi }$, can be obtained by cleaving a polymer $\Pi_{\ell }^{\tilde{S}}$ of length ℓ > L and sequence $\tilde{S}=(S,T)$ where *T* is a sequence of length ℓ−L. Similarly it can be obtained by cleaving $\Pi_{\ell }^{\tilde{S}\prime }$ of sequence $\tilde{S}\prime =(T\prime ,S)$ where T′ is also of length ℓ−L. If the rate of cleaving, $K_{\Pi }^{-}$, is assumed to be independent of the polymer length, and since there are $n^{\ell -L}$ different sequences *T* and T′, where *n* is the number of amino acid or codon *types*, the rate of concentration variation of polymers of length *L* resulting from the depolymerization of longer polymers is

(12) $$\sum_{\ell =L+1}^{L_{\text{max}}}n^{\ell -L}K_{\Pi }^{-}\;\Pi_{\ell }^{\tilde{S}}\;+\;\sum_{\ell =L+1}^{L_{\text{max}}}n^{\ell -L}K_{\Pi }^{-}\;\Pi_{\ell }^{{\tilde{S}}^{\prime }}.$$

Recall that we use the same notation for the concentration of a polymer of sequence *S* and length *L* and the polymer itself, namely $\Pi_{L}^{S}$, and Π is supposed to be set to Π=P or Π=R in our model. As already stressed, we assume polymers have at most length $L_{\text{max}}$. Finally, when the concentrations $\Pi_{L}^{\tilde{S}}$ and $\Pi_{L}^{{\tilde{S}}^{\prime }}$ are equal, (eq. 12) can be rewritten as

(13) $$2\sum_{\ell =L+1}^{L_{\text{max}}}n^{\ell -L}K_{\Pi }^{-}\;\Pi_{\ell }^{\tilde{S}}.$$

The depolymerization of polymerase $P_{L}^{\pi }$ requires special treatment. When $P_{L}^{\pi }$ depolymerizes, it generates a polymerase $P_{\ell }^{\pi }$ with ℓ<L. On the other hand, any $P_{L}^{\pi }$ can be obtained through depolymerization of one of 2*n* types of polymers of length *L* + 1, one of which being $P_{L+1}^{\pi }$ and the remaining 2n−1 being of type $P_{L+1}^{\alpha }$ with $\alpha \{L+1\}=(\pi \{L\},\alpha_{L+1}),\;\alpha_{L+1}\neq \pi_{L+1}$, or $\alpha \{L+1\}=(\alpha_{1},\;\pi \{L\})$ with *α*_1_ any of the *n* types of amino acids. More generally, they can be obtained from $P_{L+\ell^{\prime }}^{\pi }$ and $2n^{\ell^{\prime }}-1$ polymers of type $P_{L+\ell^{\prime }}^{\alpha }$ where ℓ′≥1 and $\alpha \{L+\ell \prime \}=(\pi \{L\},\;\alpha_{L+1},\ldots \alpha_{L+\ell \prime })$ with $\alpha_{j}\neq \pi_{j},j=L+1,\ldots L+\ell \prime$, or $\alpha \{L+\ell \prime \}=(\alpha_{1},\ldots \alpha_{\ell \prime },\;\pi \{L\})$ for any type $\alpha_{j},j=1,\ldots \ell \prime$. The same is true for the corresponding polynucleotide chains.

When the polymerase is bound to a polynucleotide, it becomes more stable either through induced folding of a (partially) unfolded sequence, or through the inaccessibility of bound portions, or both. We thus define $F_{\pi }(\ell )$ as the depolymerization reduction coefficient for the bound polymerase of length *ℓ*, with that reduction coefficient being 1 when no depolymerization occurs at all. We estimate it to be

(14) $$F_{\pi }(\ell )=\{\begin{array}{cc} 1-e^{-\frac{\ell -\ell_{\pi \text{min}}+1}{\lambda }} & \ell \;\geq \;\ell_{\pi \text{min}}\\ 0 & \ell \;<\;\ell_{\pi \text{min}} \end{array},$$

with λ >0 a parameter controlling how much of the polymerase is stabilized. The term $(\ell -\ell_{\pi \text{min}}+1)/\lambda$ can be interpreted as a Boltzmann factor with a free energy expressed in units of *k_BT_*. The hydrogen bond binding energy between RNA and a polypeptide is ∼16 kJ/mol [Dixit et al. 2000], so assuming that the number of such hydrogen bonds between the polymerase and the polynucleotide is $\ell -\ell_{\pi \text{min}}+1$, one has λ≈0.15.

The binding rate of a polymerase to a polynucleotide $R_{M}^{\alpha }$ of length *M* and sequence *α* is $k_{b,M}=Z\;R_{M}^{\alpha }\;n^{M}$ where *n^M^* is the total number of polynucleotides of length *M*. The probability that a polymerase of length *L* binds to a polynucleotide of length *M* is therefore given by

(15) $${\tilde{P}}_{b,M}=\frac{k_{b,M}}{\sum_{m=2}^{L_{\text{max}}}k_{b,m}}.$$

The total time the polymerase remains bound to a polynucleotide of length *M* is estimated to be $M/k_{R+}$. Therefore the probability $P_{b}$ for a polymerase to be bound is given by the average binding time divided by the sum of the average binding time and the average time needed to bind:

(16) $$P_{b}=\frac{\sum_{M=2}^{L_{\text{max}}}(M/k_{R+}){\tilde{P}}_{b,M}}{\sum_{M=2}^{L_{\text{max}}}\left((M/k_{R+}){\tilde{P}}_{b,M}\right)+1/\sum_{m=2}^{L_{\text{max}}}k_{b,m}}.$$

As a result the polymerase depolymerization rate will be

(17) $$\begin{array}{c} K_{P,\alpha ,L}^{-}=K_{P}^{-},\\ K_{P,\bar{\pi },L}^{-}=K_{P}^{-},\\ K_{P,\pi ,L}^{-}=K_{P}^{-}(1-P_{b}F_{\pi }(L)). \end{array}$$

### Equations

For any chain of length *ℓ*, our model considers the concentrations of polynucleotides and polypeptides corresponding to the polymerase sequence *π*, its complementary sequence $\bar{\pi }$ and the generic sequences *α*. We assume that the concentrations of polynucleotides and polypeptides of a specific length, bar the polymerase and its complementary sequence, are identical. For the chains that share the first *ℓ* elements of their sequence with those of the polymerase (or its complementary chain), and differ in all other elements, this is only an approximation, but it is nevertheless justified, as the concentrations of these polymers only differ slightly from those of polymers with sequences of type α, and their contribution to the variation of the polymerase concentration is expected to be small.

The variations in polymer concentrations as time evolves are governed in our model by a system of ordinary differential equations. In the equations, *L* is the length of the polymer chains, spanning all values in the range $1<L\leq L_{\text{max}}$ where $L_{\text{max}}$ is the maximal length of polypeptide and polynucleotide chains. We thus have a system of $6\times (L_{\text{max}}-1)$ equations. We recall that *n* is the number of codon types, assumed to be equal to the number of amino acid types.

$$\frac{dR_{L}^{\pi }}{dt}=K_{R}^{+}R_{1}R_{L-1}^{\pi }-nK_{R}^{+}R_{1}R_{L}^{\pi }+\sum_{\ell =L+1}^{L_{\text{max}}}\left[K_{R}^{-}R_{\ell }^{\pi }+(2n^{\ell -L}-1)K_{R}^{-}R_{\ell }^{\alpha }\right]-(L-1)K_{R}^{-}R_{L}^{\pi }+K(L)R_{L}^{\bar{\pi }},$$

$$\frac{dR_{L}^{\bar{\pi }}}{dt}=K_{R}^{+}R_{1}R_{L-1}^{\bar{\pi }}-nK_{R}^{+}R_{1}R_{L}^{\bar{\pi }}+\sum_{\ell =L+1}^{L_{\text{max}}}\left[K_{R}^{-}R_{\ell }^{\bar{\pi }}+(2n^{\ell -L}-1)K_{R}^{-}R_{\ell }^{\alpha }\right]-(L-1)K_{R}^{-}R_{L}^{\bar{\pi }}+K(L)R_{L}^{\pi },$$

$$\frac{dR_{L}^{\alpha }}{dt}=K_{R}^{+}R_{1}R_{L-1}^{\alpha }-nK_{R}^{+}R_{1}R_{L}^{\alpha }+2\sum_{\ell =L+1}^{L_{\text{max}}}n^{\ell -L}K_{R}^{-}R_{\ell }^{\alpha }-(L-1)K_{R}^{-}R_{L}^{\alpha }+K(L)R_{L}^{\alpha },$$

$$\frac{dP_{L}^{\pi }}{dt}=K_{P}^{+}P_{1}P_{L-1}^{\pi }-nK_{P}^{+}P_{1}P_{L}^{\pi }+\sum_{\ell =L+1}^{L_{\text{max}}}[K_{P}^{-}(1-P_{b}\;F_{\pi }(L))P_{\ell }^{\pi }+(2n^{\ell -L}-1)K_{P}^{-}P_{\ell }^{\alpha }]-(L-1)K_{P}^{-}(1-P_{b}\;F_{\pi }(L)P_{L}^{\pi }+k_{P,L}^{+}P_{1}R_{L}^{\pi },$$

$$\frac{dP_{L}^{\bar{\pi }}}{dt}=K_{P}^{+}P_{1}P_{L-1}^{\bar{\pi }}-nK_{P}^{+}P_{1}P_{L}^{\bar{\pi }}+\sum_{\ell =L+1}^{L_{\text{max}}}\left[K_{P}^{-}P_{\ell }^{\bar{\pi }}+(2n^{\ell -L}-1)K_{P}^{-}P_{\ell }^{\alpha }\right]-(L-1)K_{P}^{-}P_{L}^{\bar{\pi }}+k_{P,L}^{+}P_{1}R_{L}^{\bar{\pi }},$$

(18) $$\frac{dP_{L}^{\alpha }}{dt}=K_{P}^{+}P_{1}P_{L-1}^{\alpha }-nK_{P}^{+}P_{1}P_{L}^{\alpha }+2\sum_{\ell =L+1}^{L_{\text{max}}}n^{\ell -L}K_{P}^{-}P_{\ell }^{\alpha }-(L-1)K_{P}^{-}P_{L}^{\alpha }+k_{P,L}^{+}P_{1}R_{L}^{\alpha }.$$

Alongside the seven physical parameters $\left\{K_{R}^{\pm },\;K_{P}^{\pm }\;,h_{P,L}^{+},\;K(L),\;P_{b}\right\}$ appearing in the differential equations above, we need to consider two parameters yielding the “initial” concentrations of amino acid and nucleotide inside the system, namely $\rho_{p}\equiv P_{1}(t=0)$ and $\rho_{r}\equiv R_{1}(t=0)$. In the absence of actual data for these quantities, we explore a range of realistic values in the analysis of our model. The concentration of free amino acids and nucleotides at any one time is then given by $P_{1}(t)=\rho_{p}-\sum_{L=2}^{L_{\text{max}}}\left[(n^{L}-2)P_{L}^{\alpha }(t)+P_{L}^{\pi }(t)+P_{L}^{\bar{\pi }}(t)\right]$ and $R_{1}(t)=\rho_{r}-\sum_{L=2}^{L_{\text{max}}}\left[(n^{L}-2)R_{L}^{\alpha }(t)+R_{L}^{\pi }(t)+R_{L}^{\bar{\pi }}(t)\right]$ respectively, with $P_{L}^{S}(0)=R_{L}^{S}(0)=0$ for any value of *L* in the range $2\leq L\leq L_{\text{max}}$ and sequence $S=\alpha ,\pi ,\bar{\pi }$.

## Results

The system of equations (eq. 18) is nonlinear and too complex to solve analytically. We therefore analyze it numerically, starting from a system made entirely of free nucleotides, amino acids, as well as charged p-tRNA, and letting the system evolve until it settles into a steady configuration.

The main quantities of interest are the relative concentrations of the polymerase ($\rho_{\pi }$) and of the *α* peptide chains ($\rho_{\alpha }$). We have

(19) $$\rho_{\pi }=\sum_{\ell =\ell_{\pi \text{min}}}^{L_{\text{max}}}P_{\ell }^{\pi }\text{ and }\rho_{\alpha }=\sum_{\ell =\ell_{\pi \text{min}}}^{L_{\text{max}}}P_{\ell }^{\alpha },$$

and evaluate the ratios

(20) $$Q_{1}=\frac{\rho_{\pi }}{\rho_{\alpha }}\text{ and }Q_{2,\ell }=\frac{P_{\ell }^{\pi }}{P_{\ell }^{\alpha }},$$

while monitoring the evolution of each quantity over time. *Q*_1_ corresponds to the relative amount of polymerase of any length compared with other proteins (for a specific arbitrary sequence α), while $Q_{2,\ell }$ corresponds to the relative amount of polymerase of length *ℓ* compared with an arbitrary protein of length *ℓ*. Unit ratios indicate that the polymerase has not been selected at all, whereas large values of *Q*_1_ or $Q_{2,\ell }$ on the other hand indicate a good selection of the polymerase.

The complexity of the system (eq. 18) also lies in the number of free parameters it involves. A systematic analysis of the high-dimensional parameter space is beyond the scope of this article, and we therefore concentrate on the analysis and description of results for a selection of parameter values that highlight potentially interesting behaviors of our model.

Recall that our model assumes that the number *n* of different amino acids is equal to the number of codon types, and throughout our numerical work we have set *n* = 4. Note that the word “codon” here is used by extension. Indeed, there are four different nucleic acids in our model and the “biological” codons are made of two nucleic acids, bringing their number to sixteen. However, they split into four groups of four, each of which encoding one of the four amino acids. From a mathematical modeling point of view, this is completely equivalent. It is well accepted that early proteins were produced using a reduced set of amino acids (Angyan et al. 2014). The exact identity and number is unclear though experimental work has shown that protein domains can be made using predominantly five amino acids (Riddle et al. 1997) whereas the helices of a four-alpha helix bundle were made using only four amino acids (Regan and DeGrado 1988). We have used mostly $\ell_{\pi \text{min}}=7$ and $\ell_{\pi \text{max}}=L_{\text{max}}=10$, but have investigated other values as well (see the Supplementary Material online).

While these figures are somewhat arbitrary, an $\ell_{\pi \text{min}}$ of 7 was chosen on the assumption that the functional p-Pol would have some forms of stable structural motif and this number corresponds to the typical minimum number of amino acids required to produce a stable, folded alpha helix structure (Manning et al. 1988). The choice of $L_{\text{max}}=10$ is based on the fact that while the polymer peptide chains could be significantly longer, they would need correspondingly long polynucleotide sequences to encode them, which becomes increasingly unlikely as lengths increase. Furthermore, we expected polymers of length 10 to have very low concentrations, a hypothesis confirmed by our simulations. We have nevertheless investigated larger values of $L_{\text{max}}$ as well, and found little difference, as outlined below.

In a first step, guided by data on parameter values gleaned from the literature and gathered in the Supplementary Material online, we set

(21) $$\begin{array}{c} K_{R}^{+}=4.2\times 10^{-7}\;{\text{mol}}^{-1}\;m^{3}\;s^{-1},\\ K_{R}^{-}=8\times 10^{-9}s^{-1},\\ K_{P}^{+}=2.8\times 10^{-21}\;{\text{mol}}^{-1}\;m^{3}\;s^{-1},\\ K_{P}^{-}=4\times 10^{-11}s^{-1}\\ k_{P,1}^{+}=0.1\;{\text{mol}}^{-1}\;m^{3}\;s^{-1},\\ h_{R}=10^{6}\;{\text{mol}}^{-1}\;m^{3}\;s^{-1},\\ Z=10^{6}\;{\text{mol}}^{-1}\;m^{3}\;s^{-1},\\ \lambda =0.15,\\ k_{\text{step}}=4\times \;10^{-5}s^{-1}. \end{array}$$

We let the system evolve under a variety of initial concentrations of free amino acids and nucleotides, *ρ_p_* and *ρ_r_*, in the range $10^{-5}-0.1\;\text{mol}\;m^{-3}$, and with all polymer concentrations set to 0. We monitored the concentration of all polymers, in particular the concentration of polymerase $\rho_{\pi }$ and its ratio to the concentration of *α* polypeptide chains, *Q*_1_. In most cases we found that the nucleotides polymerized spontaneously (Mechanism (A)) in small amount and this led, indirectly, to the polymerization of the polypeptides, including the polymerases (Mechanism (C)). The polymerases then induced further polymerization of the polynucleotides (Mechanism (B)) and the system slowly equilibrated.

**Fig. 5.**
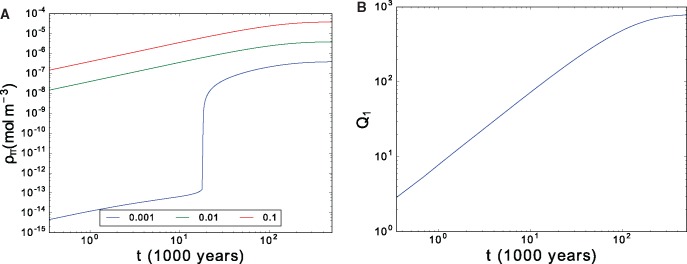
(*a*) Time evolution of the polymerase for initial concentration $\rho_{r}=\rho_{p}=0.001$, 0.01, and $0.1\;\text{mol}\;m^{-3}$. (*b*) *Q*_1_ for initial concentration $\rho_{r}=\rho_{r}=0.01\;{\text{molm}}^{-3}$. Parameter values: $K_{P}^{-}=4\times 10^{-11}s^{-1},\;Z=10^{6}\;{\text{mol}}^{-1}\;m^{3}\;s^{-1},\;\lambda =0.15$.

The end result was an excess of polymerase of all lengths compared with *α* polypeptide chains with $Q_{1}=786$ for all initial concentrations $\rho_{p}=\rho_{r}\geq 0.001\;\text{mol}\;\;m^{-3}$ (fig. 5). Moreover the total amount of polymerase reached, for initial concentration of free amino acids *ρ_p_*, was a concentration of $∼4\times \;10^{-4}\times \;\rho_{p}$ (as illustrated by the bottom two rows in table 1). The concentration of polymerase of length 10, on the other hand, was very small $P_{10}^{\pi }=6.3\times 10^{-14}\text{mol}\;\;m^{-3}$ for but $Q_{2,10}=5.9\times 10^{18}$ was very large, effectively showing that the only polypeptide chain of length $L_{\text{max}}=10$ was the polymerase.

**Table 1.** Effect of Initial Concentrations on Polymerase Production.

$\rho_{p}\;(\text{mol}\;\;m^{-3})$	1. $\rho_{r}\;(\text{mol}\;\;m^{-3})$	2. $\rho_{\pi }\;\;(\text{mol}\;\;m^{-3})$	3. *Q*_1_	4. Polymerase Production
2 × 10^–4^	2 × 10^–4^	2.8 10^–19^	1.0008	Insignificant
9 × 10^–4^	9 × 10^–4^	1.410^–14^	12.4	Insignificant
10^–3^	10^–3^	3.9 × 10^–7^	786	Yes
10^–1^	10^–1^	3.9 × 10^–5^	786	Yes

We found hardly any polymerization of the polymerase when $\rho_{p}=\rho_{r}=0.0009\;\;\text{mol}\;\;m^{-3}$, with $\rho_{\pi }\approx 1.4\times 10^{-14}\;\text{mol}\;\;m^{-3}$ and $Q_{1}=12.4$, whereas with $\rho_{p}=\rho_{r}=0.001\;\text{mol}\;m^{-3}$, we obtained $\rho_{\pi }\approx 3.9\times 10^{-7}\;\text{mol}\;\;m^{-3}$ and $Q_{1}=786$ (fig. 5*a*). This highlights a very sharp transition at a *critical concentration*$\rho_{p,c}$ above which polymerases are generated. We summarize the data in table 1.

We then fixed the initial concentration *ρ_p_* to four different values and varied *ρ_r_* to identify the critical initial concentration of nucleotides necessary for the production of polymerases. The results in table 2 show that the critical concentration $\rho_{r,c}$ is nearly constant and of the order of $10^{-3}\;\text{mol}\;\;m^{-3}$ for a very wide range of amino acid initial concentrations.

**Table 2.** Effect of Initial Peptide Concentration on Critical Concentration.

$\rho_{p}\;(\text{mol}\;\;m^{-3})$	$\rho_{r,c}\;(\text{mol}\;\;m^{-3})$
10^–4^	2 × 10^–3^
10^–3^	10^–3^
10^–2^	8 × 10^–4^
10^–1^	7 × 10^–4^

Many of the parameters we have used were estimated or measured in conditions which, in all likelihood, were not identical to the ones existing when the polymerization we are modeling occurred. In a second step, we departed from the set of values (eq. 21) and found that in all cases investigated, varying these parameters modified the critical concentrations of $\rho_{r,c}$ and $\rho_{p,c}$, but did not affect significantly the value of *Q*_1_ while $Q_{2,10}$ remained extremely large.

More specifically, taking $K_{P}^{-}=5.1\times 10^{-6}s^{-1}$ marginally increased the critical concentration to $\rho_{r,c}=\rho_{p,c}=0.0011\;\text{mol}\;\;m^{-3}$. Similarly, taking $k_{\text{step}}=0.02s^{-1}$ increased slightly the critical concentrations: $\rho_{r,c}=\rho_{p,c}=0.0017\;\text{mol}\;\;m^{-3}$. On the other hand, taking $Z=10^{8}{\text{mol}}^{-1}\;m^{3}\;s^{-1}$ lead to a decrease of the critical concentrations: $\rho_{r,c}=\rho_{p,c}=0.0005\;\text{mol}\;\;m^{-3}$. Varying *h_R_* to values as small as $1\;{\text{mol}}^{-1}\;m^{3}\;s^{-1}$ did not change the critical concentrations.

In our model, we have considered the concentrations of free amino acids ($\rho_{p}\equiv P_{1}$) and charged p-tRNA to be identical: $k_{t}\approx 1$ (see eq. 7). To consider other values of *k_t_* we only need to multiply the polymerization rate of a peptide ($k_{P,1}^{+}$) by *k_t_* as it is p-tRNAs that bind to XNA chains, not free amino acids. We have considered a large range of values for $k_{P,1}^{+}$ and found that for $k_{P,1}^{+}=10^{-5}\;{\text{mol}}^{-1}\;m^{3}\;s^{-1}$, the critical concentrations had not changed significantly while for $10^{-8}\;{\text{mol}}^{-1}\;m^{3}\;s^{-1}$, they increased to $\rho_{r,c}=\rho_{p,c}=0.002\;\text{mol}\;\;m^{-3}$. This shows that taking much smaller values of *k_t_* has a very small impact on our results and that having a concentration of charged p-tRNA much smaller than that of free amino acids would only increase marginally the critical concentrations we have obtained using our original assumption.

The parameters on which the model is the most sensitive are $K_{R}^{+}$ and $K_{R}^{-}$. We found that for $K_{R}^{+}=4\times 10^{-8}\;{\text{mol}}^{-1}\;m^{3}\;s^{-1}$, $\rho_{r,c}=\rho_{p,c}=0.007\;\text{mol}\;\;m^{-3}$ and for $K_{R}^{+}=4\times 10^{-9}\;{\text{mol}}^{-1}\;m^{3}\;s^{-1}$, $\rho_{r,c}=\rho_{p,c}=0.05\;\text{mol}\;\;m^{-3}$. Similarly, for $K_{R}^{-}=10^{-7}\;s^{-1}$ we found that $\rho_{r,c}=\rho_{p,c}=0.01\;\text{mol}\;\;m^{-3}$ and for $K_{R}^{-}=10^{-6}\;s^{-1}$ that $\rho_{r,c}=\rho_{p,c}\approx 0.05\;\text{mol}\;\;m^{-3}$. This shows that the spontaneous polymerization of polynucleotide is essential to reach a minimum concentration of polynucleotides to kick start the whole catalysis process and that the stability of the polynucleotides plays an important role.

To investigated this, we have run simulations with $K_{R}^{+}=4\times 10^{-8}\;{\text{mol}}^{-1}\;m^{3}\;s^{-1}$ for a fixed duration, $\tau_{\text{pol}}$, after which $K_{R}^{+}$ was set to 0. We found that if $\tau_{\text{pol}}$ was long enough, the polymerization of polypeptide and polynucleotide chains was identical to the one obtained whereas $K_{R}^{+}$ was not modified. When $\tau_{\text{pol}}$ was too short, on the other hand, one was only left with short polypeptide and polynucleotide chains in an equilibrium controlled by the spontaneous polymerization and depolymerization parameters. The minimum value for $\tau_{\text{pol}}$ depends on the concentrations *ρ_r_* and *ρ_p_* and the results are given in table 3.

**Table 3.** Effect of Initial Concentration of Free Nucleotides on Time for Production of Polymerase.

$\rho_{r}=\rho_{p}\;\;(\text{mol}\;m^{-3})$	$\tau_{\text{pol}}\;\;(years)$
0.001	18,000
0.002	254
0.005	12.7
0.01	2.2

This shows that while $K_{R}^{+}$ is an important parameter in the process, what matters are to have a spontaneous generation of polynucleotides at the onset (Mechanism (A)). This then leads to the production of polypeptides, including polymerase (Mechanism (C)) and, once the concentration of polymerase is large enough, the catalyzed production of polynucleotides (Mechanism (B)) dominates the spontaneous polymerization.

We have also varied $K_{R}^{-}$ once the system had settled and we found that for $\rho_{r}=\rho_{p}=0.01\;\text{mol}\;m^{-3},\;K_{R}^{-}$ could be increased up to $6\times 10^{-7}s^{-1}$ while still keeping a large amount of polymerase. Above that value, the polynucleotides are too unstable and one ends up again with mostly short polymer chains and $Q_{1}\approx 1$.

We have also considered values of $L_{\text{max}}>10$ and found that the main difference is a slight increase of the critical concentrations. For example, for $L_{\text{max}}=11,12$ and 15, $\rho_{r,c}=\rho_{p,c}$ are respectively equal to 0.001,0.0011, and $0.0011\;\text{mol}\;\;m^{-3}$. At given concentrations *Q*_1_ and $\rho_{\pi }$ remain unchanged but $P_{L_{\text{max}}}^{\pi }$ deceases approximately by a factor of 40 each time $L_{\text{max}}$ is increased by 1 unit.

We have also taken $L_{\pi \text{min}}=4,5$, and 6 and found that the critical concentrations were respectively $2\times 10^{-5},2\times 10^{-4}$, and $4\times 10^{-4}\;\text{mol}\;\;m^{-3}$, whereas $\rho_{\pi }$ took the values of ∼0.012, $2.6\times 10^{-3}$, and $3\times 10^{-4}\;\text{mol}\;\;m^{-3}$. *Q*_1_ on the other hand remained constant.

A summary of the parameter values investigated outside the set (eq. 21) and the corresponding critical concentrations are given in table 4. Only one parameter was changed at a time (see the Supplementary Material online).

**Table 4.** Effect of Various Parameters on Initial Critical Concentrations.

Modified Parameter	$\rho_{r,c}=\rho_{p,c}\;\;(\text{mol}\;\;m^{-3})$
$K_{P}^{-}=5.1\times 10^{-6}\;s^{-1}$	1.1 × 10^–3^
$k_{\text{step}}=2\times 10^{-2}\;s^{-1}$	1.7 × 10^–3^
$Z=10^{8}\;{\text{mol}}^{-1}\;m^{-3}s^{-1}$	5 × 10^–4^
$h_{R}=1\;{\text{mol}}^{-1}\;m^{-3}s^{-1}$	10^–3^
$k_{P,1}^{+}=10^{-5}\;{\text{mol}}^{-1}\;m^{3}\;s^{-1}$	10^–3^
$k_{P,1}^{+}=10^{-8}\;{\text{mol}}^{-1}\;m^{3}\;s^{-1}$	2 × 10^–3^
*L*_max_ = 15	1.1 × 10^–3^
$L_{\pi \text{min}}=6$	4 × 10^–4^
$L_{\pi \text{min}}=5$	2 × 10^–4^
$L_{\pi \text{min}}=4$	2 × 10^–5^
$K_{R}^{+}=4\times 10^{-8}\;{\text{mol}}^{-1}\;m^{3}\;s^{-1}$	7 × 10^–3^
$K_{R}^{+}=4\times 10^{-9}\;{\text{mol}}^{-1}\;m^{3}\;s^{-1}$	5 × 10^–2^
$K_{R}^{-}=10^{-7}\;s^{-1}$	10^–2^
$K_{R}^{-}=10^{-6}\;s^{-1}$	0.19

## Discussion

**Fig. 6.**
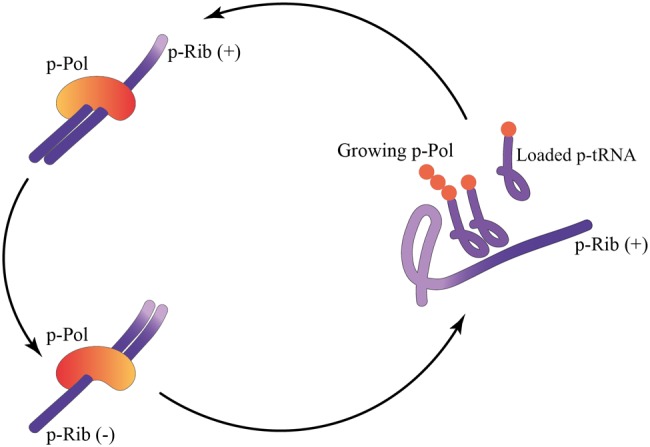
The nucleopeptide Initial Darwinian Ancestor. In this cartoon model, a short strand of XNA has the functionality of both a primordial p-mRNA and a p-Rib. Primitive XNA molecules loaded with amino acids (p-tRNA) bind to the p-Rib via codon–anticodon pairing. This allows adjacent amino acids to undergo peptide bond formation and a short peptide chain is produced. A certain peptide sequence is able to act as a primordial XNA-dependent XNA polymerase (p-Pol) able to copy both + and – p-Rib strands to eventually produce a copy of the p-Rib(+) strand.

We describe a theoretical nucleopeptidic reciprocal replicator comprising a polynucleotide that templates the assembly of small p-tRNA adapter molecules, most likely having mixed backbone architectures. These spontaneously arising p-tRNAs would have been bound to various classes of amino acids (possibly via weak stereochemical specificity), and a simple increase in local concentration mediated by binding to the p-Rib (in its most primitive version nothing much more than a mixed backbone architecture p-mRNA) could have driven polypeptide polymerization. Once a template arose that coded for a peptide able to catalyze phosphodiester bond formation, this p-Rib could have templated assembly of its own complementary strand (and vice versa) and the self-replication cycle would have been complete (see fig. 6 for a summary).

Starting from a single peptide and single polynucleotide, the IDA would quickly have become a distribution of related sequences of peptides and XNAs. We can imagine that over time, different p-Ribs encoding different peptides with additional functionalities could have appeared as the system evolved and that these p-Ribs may have subsequently fused together into larger molecules.

By imagining the IDA swiftly becoming a pool of molecules where variety within the “species” is maintained by the poor copying fidelity of a statistical operational code, should any mutation that stops replication arise, the other molecules in the pool would still function, ensuring continuity of the whole. Indeed this could have provided a selective pressure for superior replicators. While our model does not directly consider less than perfect copying fidelity, it is not expected to have a major effect on our conclusions as copies with decreased performance would not be maintained as a significant proportion of the population and copies with increased performance would simply take over the role of main replicator.

The primordial operational code may only have required two bases per p-tRNA to deliver statistical proteins, while the catalytic requirements of the p-Pol are loose enough that a seven-residue peptide is a plausible lower length limit. This reduces the minimum length of the posited spontaneously arising p-Rib to just 14 nucleotides (assuming no spaces between codons). This is an optimistic length estimate, but given the available time and with molecular co-evolution, inorganic catalysts and geological PCR, considerably longer molecules may have been possible (Baaske et al. 2007; Fishkis 2011). These p-Pols would act on p-Ribs and the crucial abiogenesis step would be the emergence of a 14-mer XNA that, in the context of the primordial operational code, happened to code for a peptide able to bind XNA and catalyze phosphodiester bond formation of base-paired nucleotides. Although the concentrations of various components are not known with certainty this does not seem an unreasonable proposition particularly given that functional peptides are known to occur in random sequences with surprising frequency (Keefe and Szostak 2001).

Our mathematical model showed that the most important parameters, apart from the concentration of loaded p-tRNA and polynucleotides, are the spontaneous polymerization and depolymerization of polynucleotides. It also shows that polynucleotides are first polymerized spontaneously and that these initial polynucleotides catalyze the production of the first polypeptides, including the polymerase. These polymerases can then generate further polynucleotides through catalysis. The stability gained by polymerases while being bound to polynucleotides ultimately leads to an increase of their relative concentration compared with the other polypeptides.

Overall, the hypothesis explains the coupling of polynucleotide and polypeptide polymerization, the operational code and mutations in the p-Pol sequence that could eventually result in increased specificities leading to primitive DNA polymerases and RNA polymerases. No extraordinary exchanges of function are required and each molecule is functionally similar to its present-day analogue. Like all new abiogenesis theories, this IDA requires in vitro confirmation; in particular, the steps required for the primordial operational code to arise ab initio warrant close attention.

The idea that the ancestral replicator may have consisted of both nucleic acid and peptide components (the “nucleopeptide world”) is in itself not new, but compared with the RNA world, has been somewhat neglected. We argue that molecular co-evolution of polynucleotides and peptides seems likely and cross-catalysis is known to be possible, for example in vitro selection experiments delivered RNA with peptidyl transferase activity after just nine rounds of a single selection experiments (Zhang and Cech 1997; Fishkis 2011). Inversely, Levy and Ellington produced a 17-residue peptide that ligates a 35 base RNA (Levy and Ellington 2003).

Nucleopeptide world research is relatively sparse, the data collected so far hint that cross-catalysis may be more efficient than autocatalysis by either peptides or nucleic acids. A self-replicating primordial system wherein RNA encoding for protein was replicated by a primordial RNA-dependent RNA polymerase which carried out the role of a replicative agent rather than as a transcriber of genes has previously been suggested (Leipe et al. 1999), although in this case no further development of the concept to produce a self-contained replicating system was pursued. The merits of a “two polymerase” system where RNA catalyses peptide polymerization and vice versa were succinctly explained by Kunin (2000), although possible mechanisms and validity were not considered in detail. The possibility of a two polymerase system is also mentioned by van der Gulik and Speijer as part of a wider review of the co-evolution of peptides and RNA (van der Gulik and Speijer 2015) but without a mathematical model.

Other origins of life hypotheses propose that the initial self-replicator did not consist of polynucleotides and/or peptides but was originally composed of different materials, most famously clay crystals (Cairns-Smith 1982). Such hypotheses are of interest but were not considered in this work as the IDA presented here does not require genetic takeover of one replication system by another and can be achieved using building blocks likely to have been present on the early earth and so appears more parsimonious. Our IDA hypothesis has tried to set out more rigorously the possible steps and processes whereby a nucleopeptide IDA could have arisen and could be tested experimentally.

Future experimental work that would support the nucleopeptide theory would be to provide evidence that the stereochemical hypothesis applies to the earliest occurring amino acids including those likely to have composed the active site of the p-Pol. Currently codon/anticodon binding to a number of amino acids has been shown (Yarus et al. 2005) but is absent for the four earliest amino acids (Wolf and Koonin 2007). This may be due to their small sizes though even here possible solutions have been proposed (Tamura 2015).

It is important to note that we do not propose that the RNA world did not or could not exist, nor does this work necessarily suggest that a self-replicating RNA polymerase did not exist (although our results suggest it to be unlikely), but rather that such a molecule did not directly lead to current living systems. Indeed the crucial role of RNA (more correctly, XNA) in our model is highlighted by the importance of $K_{R}^{+}$, the rate of polymerization of polynucleotide chains. We also do not dismiss any roles for ribozymes—for example it could well be that ribozymes were responsible for aminoacylation reactions (although this would inevitably raise the question of how such ribozymes were themselves replicated). Similarly (and with similar provisos), peptides alone could also have carried out supporting roles such as stabilizing long XNA sequences or catalyzing aminoacylation reactions. At its core however, we suggest that the ancestral replicator was nucleopeptidic with information storage function carried out by the XNA and polymerase function carried out by the peptide.

## Supplementary Material

Supplementary data are available at *Molecular Biology and Evolution* online.

## Supplementary Material

Supplementary Data
