# Supplementary material for: Reciprocal Nucleopeptides as the Ancestral Darwinian Self-Replicator
Source: Mol Biol Evol. 2017 Nov 8;35(2):404–16. doi: 10.1093/molbev/msx292 (PMC5850689; doi:10.1093/molbev/msx292)
Supplement: Supplementary Data [file msx292_suplementarymat_v3.pdf]

Article, Discoveries

Reciprocal Nucleopeptides as the Ancestral Darwinian Self-Replicator

Eleanor F. Banwell[1*], Bernard M.A.G. Piette[2*], Anne Taormina[2] and Jonathan G. Heddle[1,3,♮]

[1] RIKEN, Hirosawa 2-1, Wako-shi, Saitama, 351-0198 Japan

[2] Department for Mathematical Sciences, Durham University, South Road, Durham DH1 3LE, United Kingdom

[3] Bionanoscience and Biochemistry Laboratory, Malopolska Centre of Biotechnology, Jagiellonian University 30-387, Krakow, Poland.

[*] These authors contributed equally

[♮] *Corresponding author:* Heddle, J.G. (jonathan.heddle@uj.edu.pl)

1 Supplementary Information

In this section we use the SI units metres (m), seconds (s), kilogrammes (kg) and moles (mol). The mole/litre (molar M) is thus expressed as $\mathrm{M} = 10^3 \mathrm{mol/m}^3$ in SI units.

1.1 Evaluation of the model parameter values

Peptide hydrolysis rate

The hydrolysis rate of a peptide bond in neutral water has been measured as $3 \times 10^{-9}\,\mathrm{s}^{-1}$, i.e. about 7 years (Kahne and Still 1988).

Hydrolysis rate of an internal peptide bond was estimated to be $3.6 \times 10^{-11}\,\mathrm{s}^{-1}$ at 25° C, $1.13 \times 10^{-9}\,\mathrm{s}^{-1}$ at 37° C, $1 \times 10^{-7}\,\mathrm{s}^{-1}$ at 95° C , $1 \times 10^{-8}\,\mathrm{s}^{-1}$ at 60° C and $5.1 \times 10^{-6}\,\mathrm{s}^{-1}$ at 150° C and to be relatively insensitive to pH in the range $4.2 - 7.8$ (Radzicka and Wolfenden 1996; Wolfenden 2014).

So we can use values of K_P^- in the range

$$K_P^- :\ 4 \times 10^{-11}\,\mathrm{s}^{-1} - 5.1 \times 10^{-6}\,\mathrm{s}^{-1}.$$

RNA hydrolysis rate

DNA hydrolysis rate is pH independent around neutral pH and in that case cleavage of phosphodiester linkages have a half life of 140000 years (i.e. $1.6 \times 10^{-13}\,\mathrm{s}^{-1}$ assuming 1st order kinetics at 25°C) and 22 years at 100°C. RNA is much less stable, its half life being 4 years at 25° C and 9 days at 100° C (Wolfenden and Snider 2001). RNA is more stable at pH 4-6 compared to higher pH (Bernhardt and Tate 2012) but we assume this to be a relatively moderate effect for our suggested pH 5 and so retain these numbers i.e. 4 years. $\approx 8 \times 10^{-9}\,\mathrm{s}^{-1}$. We actually suggest that the system originally was XNA, possibly a mix of RNA-DNA and related molecules, meaning that stability was likely higher. But for simplicity it seems reasonable to keep this estimate of

$$K_R^- \approx 8 \times 10^{-9}\,\mathrm{s}^{-1}.$$

Polypeptide and Polynucleotide Spontaneous Polymerisation

Many of the parameters in our model are unknown, but we can try to estimate them using simple kinetic theory. This will give some estimates or upper bound on the values we should use.

In a perfect gas, the number of collisions per unit volume between two molecules A and B is given by

$$Z_{A,B} = \rho_A \rho_B (r_A + r_B)^2 \sqrt{\frac{8\pi \mathrm{k_B T}}{\mu_{AB}}}$$

where ρ_A and ρ_B are the concentration of each reactant, r_A and r_B the radius of the molecules, $\mathrm{k_B}$ the Boltzmann constant, T the temperature in Kelvin and $\mu_{AB} = \mathrm{m}_A \mathrm{m}_B/(\mathrm{m}_A + \mathrm{m}_B)$ their reduced mass. If $\mathrm{m}_A >> \mathrm{m}_B$ then $\mu_{AB} \approx \mathrm{m}_B$.

If the reaction is $A + B \to C$, the equation we have to solve is

$$\frac{d\rho_C}{dt} = \rho_A \rho_B (r_A + r_B)^2 \sqrt{\frac{8\pi \mathrm{k_B T}}{\mu_{AB}}} K_{A,B}$$

where $K_{A,B} < 1$ includes the activation factor. If ρ_C and ρ_B can be expressed in any units, ρ_A must be expressed as the number of molecules per unit volume. If we express the densities in $\mathrm{mol\,m^{-3}}$, we thus have

$$\frac{d\rho_C}{dt} = \rho_A\, \rho_B\, z_{A,B}\, K_{A,B}$$

where

$$z_{A,B} = N_a(r_A + r_B)^2 \sqrt{\frac{8\pi k_B T}{\mu_{AB}}}$$

is the quantity one needs to estimate.

For a nucleotide and amino acids we have: $r_{\text{nuc}} \approx 5.5$Å (Hyeon 2006), $m_{\text{nuc}} \approx 500\text{g/mol} = 500\text{g}/6 \times 10^{23} \approx 8.3 \times 10^{-25}\text{kg}$, $r_{\text{am}} \approx 5$Å and $m_{\text{am}} \approx 100\text{g/mol} = 100g/6, 10^{23} \approx 1.7 \times 10^{-25}\text{kg}$ We also have $\mathrm{k_B} = 1.38 \times 10^{-23}\mathrm{JK}^{-1}$, $\mathrm{T} = 300\mathrm{K}$ so $\mathrm{k_B T} \approx 4 \times 10^{-21}\mathrm{J}$ and Avogadro's number $\mathrm{N}_a \approx 6\ \times 10^{23}$.

So the collision rate of two nucleotides can be estimated as

$$z_{n,n} \approx \mathrm{N}_a(2r_{\text{nuc}})^2 \sqrt{\frac{8\pi \mathrm{k_B T}}{\mathrm{m_{nuc}}/2}} \approx 6.6 \times 10^8\,\mathrm{s}^{-1}\ \mathrm{mol}^{-1}\,\mathrm{m}^3.$$

Moreover, the uncatalysed phosphodiester bond formation in solution is thought to have an activation energy E_a of 21.1 kcal/mol $\approx\ 35\mathrm{k_B T}$ (Florian et al 2003). So

$$K_R^+ = 6.6 \times 10^8\,\mathrm{mol}^{-1}\,\mathrm{m}^3 \times \mathrm{e}^{-35} \approx 4.2 \times 10^{-7}\,\mathrm{mol}^{-1}\,\mathrm{m}^3.$$

The collision rate of 2 polypeptides can be estimated as

$$z_{a,a} \approx N_a(2r_{\text{am}})^2 \sqrt{\frac{8\pi k_B T}{m_{\text{am}}/2}} \approx 3.5 \times 10^8\,\mathrm{s}^{-1}\,\mathrm{mol}^{-1}\,\mathrm{m}^3.$$

For the activation energy of polypeptide chains we take $E_{a,R} = 40\,\text{kcal/mol} = 67\mathrm{k_B T}$ so

$$K_P^+ = 3.5 \times 10^8\,\mathrm{mol}^{-1}\,\mathrm{m}^3 \times e^{-67} \approx 2.8 \times 10^{-21}\,\mathrm{mol}^{-1}\,\mathrm{m}^3.$$

Similarly, we can estimate the collision rate between a polynucleotide of length L and a polymerase of length ℓ to be

$$z_{\ell,L} \approx \mathrm{N}_a(\sqrt{L}r_{\text{nuc}} + \sqrt{l}r_{\text{am}})^2 \sqrt{\frac{8\pi \mathrm{k_B T}}{\mu_{L,\ell}}},$$

where $\mu_{L,\ell} = L\ell m_{\text{nu}} m_{\text{am}}/(L m_{\text{nu}} + \ell m_{\text{am}})$. For $L = \ell = 10$ we have $z_{\ell,L} \approx 1.5 \times 10^9\mathrm{s}^{-1}\,\mathrm{mol}^{-1}\,\mathrm{m}^3$. We then have

$$Z_{\ell,L} \approx z_{L,l} K_{L,l}$$

where $K_{L,l}$ is an activation factor which we have conservatively estimated to be 1/1500. Moreover as all the polymerases and catalysed polypeptide chains do not vary much in length we can assume $Z_{\ell,L}$ to be independent of ℓ and L and so $Z = Z_{\ell,L} \approx 10^{-6}\mathrm{s}^{-1}\,\mathrm{mol}^{-1}\,\mathrm{m}^3$.

RNA polymerisation rate (with concentrations)

Experiments have been carried out with the concentration of primordial polymerase (in this case an RNA molecule) present at a concentration of $2\,\mu M$, a template strand, present at $1\,\mu$M and a small primer (this is the RNA strand that will be extended, bound to the template) at $0.5\,\mu$M (Lawrence and Bartel 2003). The material to be added to the end of the primer, i.e. activated nucleotide triphosphates, were present at large excess, i.e. $100\,\mu M$. It was found that the polynucleotide elongates by one units at a rate varying between $0.02\,\mathrm{s}^{-1}$ and $4\times10^{-5}\,\mathrm{s}^{-1}$. So we have

$$2\times10^{-2}\,\mathrm{s}^{-1} \leq k_{\mathrm{step}} \leq 4\times10^{-5}\mathrm{s}^{-1}.$$

h_R can be estimated from the collision rate of 2 nucleotides which we evaluated above to be of the order $6.6\times10^{8}\,\mathrm{s}^{-1}\,\mathrm{mol}^{-1}\,\mathrm{m}^{3}$, but we must add to that a factor taking into account the correct orientation of the nucleotide and so we will take as an estimate

$$h_R = 10^{6}\,\mathrm{s}^{-1}\,\mathrm{mol}^{-1}\,\mathrm{m}^{3}.$$

The actual value does not matter much as the limiting factor in the duplication of the polynucleotide is the rate k_{step}.

Peptide polymerisation rate

It is estimated (Wohlgemuth et al 2006) that the rate of peptide bond formation is approximately $0.001\,\mathrm{s}^{-1}$ under the following conditions: at 50 mM Mg^{2+}, with the concentration of the primitive ribosome (50S subunits) at $0.6\,\mu M$, the concentration of tRNA carrying an amino acid (fMet-tRNA) at $6.6\,\mu$M and the concentration of the peptide acceptor (puromycin) at 10 mM (to ensure complete saturation of the ribosome). However, it could be as small as $10^{-8}\,\mathrm{s}^{-1}$ (Sievers et al 2004).

This suggests we take $k^{+}_{P,L}\,P_1$ in the range $10^{-7}\,\mathrm{s}^{-1} - 10^{-3}\,\mathrm{s}^{-1}$ with $P_1 \approx 10^{-5}M$, that is $k^{+}_{P,L}$ in the range $10^{-5}\,\mathrm{s}^{-1}\,\mathrm{mol}^{-1}\,\mathrm{m}^{3}$ - $10^{-1}\,\mathrm{s}^{-1}\,\mathrm{mol}^{-1}\,\mathrm{m}^{3}$ and so $k^{+}_{P,1} \approx Lk^{+}_{P,L}$ in the range $10^{-4}\,\mathrm{s}^{-1}\,\mathrm{mol}^{-1}\,\mathrm{m}^{3}$ - $1\,\mathrm{s}^{-1}\,\mathrm{mol}^{-1}\,\mathrm{m}^{3}$.

We have used mostly $k^{+}_{P,1} = 0.1\,\mathrm{s}^{-1}\,\mathrm{mol}^{-1}\,\mathrm{m}^{3}$, but we also considered smaller values.

1.2 Model parameter scanning

Most parameters in our model had to be estimated or inferred from known measured values. To test the robustness of our model we have thus solved the mathematical equations for a large range of parameter values around the estimated values and we present a more detailed description of the results in this section.

The standard value of the parameters used in the paper are the following:

$$\begin{array}{lll} K_R^+ = 4.2 \times 10^{-7}\,\mathrm{mol}^{-1}\,\mathrm{m}^3\,\mathrm{s}^{-1}, & K_R^- = 8 \times 10^{-9}\mathrm{s}^{-1}, & K_P^+ = 2.8 \times 10^{-21}\,\mathrm{mol}^{-1}\,\mathrm{m}^3\,\mathrm{s}^{-1}, \\ K_P^- = 4 \times 10^{-11}\mathrm{s}^{-1}, & k_{P,1}^+ = 0.1\,\mathrm{mol}^{-1}\,\mathrm{m}^3\,\mathrm{s}^{-1}, & h_R = 10^6\,\mathrm{mol}^{-1}\,\mathrm{m}^3\,\mathrm{s}^{-1}, \\ Z = 10^6\,\mathrm{mol}^{-1}\,\mathrm{m}^3\,\mathrm{s}^{-1}, & \lambda = 0.15, & L_{max} = 10, \\ l_{\pi\,min} = 7, & k_{\mathrm{step}} = 4 \times\ 10^{-5}\mathrm{s}^{-1} & \end{array} \quad (1)$$

and these are the values we have used except when specified otherwise.

Before we scan parameters, we present the values of the concentrations of the polymers of different length after the system has settled to a constant configuration. The table below contains the concentrations obtained for the parameters given above and for the initial concentration $\rho_p = \rho_r = 0.001\mathrm{mol}\,\mathrm{m}^{-3}$. The first column corresponds to the length of the polymer. One sees that up to $L = 6$, the concentrations of all the polypeptide chains are identical because the polymerases are not active for such short length ($l_{\pi\,min} = 7$). As the length increases the concentration of the polymerases of a given length becomes orders of magnitude larger than that of other polypeptides of the same length.

L	P_L^π	$P_L^{\bar\pi}$	P_L^α	$R_L^\pi = R_L^{\bar\pi} = R_L^\alpha$
2	1.98848×10^{-4}	1.98848×10^{-4}	1.98848×10^{-4}	1.52119×10^{-4}
3	4.6605×10^{-6}	4.6605×10^{-6}	4.6605×10^{-6}	9.50744×10^{-6}
4	2.4856×10^{-7}	2.4856×10^{-7}	2.4856×10^{-7}	9.50744×10^{-7}
5	1.94188×10^{-8}	1.94188×10^{-8}	1.94188×10^{-8}	1.18843×10^{-7}
6	1.90225×10^{-9}	1.90225×10^{-9}	1.90225×10^{-9}	1.69776×10^{-8}
7	3.89253×10^{-7}	4.95377×10^{-10}	4.95377×10^{-10}	1.06085×10^{-8}
8	1.34411×10^{-12}	3.35313×10^{-19}	3.35313×10^{-19}	9.57404×10^{-18}
9	1.59048×10^{-14}	1.22373×10^{-28}	1.22373×10^{-28}	4.49223×10^{-27}
10	1.38517×10^{-16}	3.16354×10^{-38}	3.16354×10^{-38}	1.45159×10^{-36}

In the following section we describe the impact of varying all the parameters above and show that our observation is very robust with respect to the variation of all the parameters. We show that the only parameters which have a significant impact are K_P^-, K_P^+ and also λ.

Variation of L_{max}

To perform our analysis, we have to chose the maximal length of the polymers for which we solved the equations of the model and we have chosen $L_{max} = 10$. We have then solved the mathematical model for larger values of L_{max} and found that it did not make a significant difference: the value of Q_1 and the critical values for ρ_p and ρ_r did not depend on L_{max}.

$L_{max} = 10$

$\rho_p = \rho_r$	Q_1
0.0005	1.29516
0.0009	12.3813
0.001	785.772
0.002	785.764
0.005	785.773
0.01	785.770
0.02	785.769
0.05	785.770
0.1	785.774

$L_{max} = 11$

$\rho_p = \rho_r$	Q_1
0.0005	1.29514
0.0009	12.1723
0.001	785.774
0.002	785.775
0.005	785.775
0.01	785.774
0.02	785.774
0.05	785.774
0.1	785.775

$L_{max} = 12$

$\rho_p = \rho_r$	Q_1
0.0005	1.29512
0.0009	12.0454
0.001	25.0395
0.002	785.775
0.005	785.775
0.01	785.774
0.02	785.774
0.05	785.774
0.1	785.773

$L_{max} = 15$

$\rho_p = \rho_r$	Q_1
0.0005	1.2951
0.0009	11.852
0.001	22.5746
0.002	785.775
0.005	785.775
0.01	785.774
0.02	785.774
0.05	785.774
0.1	785.774

$L_{max} = 20$

$\rho_p = \rho_r$	Q_1
0.0005	1.29509
0.001	21.5498
0.0011	45.581
0.0012	785.423
0.002	785.760
0.005	785.761
0.01	785.762
0.02	785.759
0.05	785.758
0.1	785.755

Variation of $l_{\pi\,\mathrm{min}}$

We have also varied the minimum length $l_{\pi\,\mathrm{min}}$ that the polymerase must have before it becomes active as a polymerase. This did not change the larger values of Q_1 but the critical concentrations ρ_p and ρ_r decreased with $l_{\pi\,\mathrm{min}}$, dropping from $\rho_p = \rho_r = 1, \times 10^{-3}\,\mathrm{mol\,m^{-3}}$ when $l_{\pi\,\mathrm{min}} = 7$ to $\rho_p = \rho_r = 2, \times 10^{-5}\,\mathrm{mol\,m^{-3}}$ when $l_{\pi\,\mathrm{min}} = 4$.

$l_{\pi\,\mathrm{min}} = 6$

$\rho_p = \rho_r$	Q_1
0.0001	1.00176
0.0002	1.09811
0.0003	1.99223
0.0004	785.772
0.001	785.773
0.002	785.773
0.005	785.773
0.01	785.773
0.02	785.773
0.05	785.774
0.1	785.774

$l_{\pi\,\mathrm{min}} = 5$

$\rho_p = \rho_r$	Q_1
5×10^{-5}	1.01195
0.0001	1.39841
0.0002	785.771
0.001	785.773
0.002	785.772
0.005	785.773
0.01	785.773
0.02	785.773
0.05	785.773
0.1	784.072

$l_{\pi\,\mathrm{min}} = 4$

$\rho_p = \rho_r$	Q_1
1×10^{-6}	1
2×10^{-6}	1.00001
5×10^{-6}	1.00039
1×10^{-5}	1.00686
2×10^{-5}	785.769
5×10^{-5}	785.772
0.0001	785.773
0.001	785.772
0.002	785.772
0.005	785.772
0.01	785.772
0.02	785.675

Variation of K_P^-

The polypeptides depolymerisation rate, K_P^-, has a very small influence on the selection process of the polymerase. As shown in the table below, increasing K_P^- to $1 \times 10^{-8}\,\mathrm{s}^{-1}$ does not change the critical concentration nor the actual selection rate Q_1. Increasing K_P^- from $1 \times 10^{-8}\,\mathrm{s}^{-1}$ to $5.1 \times 10^{-6}\,\mathrm{s}^{-1}$ only increases the critical concentration slightly from $0.001\mathrm{mol\,m}^{-3}$ to $0.0011\mathrm{mol\,m}^{-3}$.

ρ	K_P^-	Q_1
0.0009	4×10^{-11}	12.3813
0.001	4×10^{-11}	785.774
0.005	4×10^{-11}	785.775
0.01	4×10^{-11}	785.762
0.02	4×10^{-11}	785.774
0.05	4×10^{-11}	785.761
0.1	4×10^{-11}	785.774
0.001	1×10^{-8}	785.850
0.005	1×10^{-8}	785.885
0.0009	1×10^{-8}	12.3798
0.01	1×10^{-8}	785.901

ρ	K_P^-	Q_1
0.02	1×10^{-8}	785.876
0.05	1×10^{-8}	785.907
0.1	1×10^{-8}	785.977
0.001	5.1×10^{-6}	24.535
0.0011	5.1×10^{-6}	785.765
0.005	5.1×10^{-6}	785.625
0.01	5.1×10^{-6}	785.473
0.02	5.1×10^{-6}	785.311
0.05	5.1×10^{-6}	785.312
0.1	5.1×10^{-6}	784.984

Variation of K_R^+

The polymerisation of polynucleotides is essential to kick-start the polymerisation of polypeptides and so the polymerisation rate of polynucleotide K_R^+ must be sufficiently large before polymerases can be selectively generated. We found that with $K_R^+ = 4 \times 10^{-8}\,\mathrm{mol}^{-1}\,\mathrm{m}^3\,\mathrm{s}^{-1}$ the critical concentration of ρ_p and ρ_r is $0.007\,\mathrm{mol\,m}^{-3}$ and for $K_R^+ = 4 \times 10^{-9}\,\mathrm{mol}^{-1}\,\mathrm{m}^3\,\mathrm{s}^{-1}$ it

is 0.045 mol m^{-3} while for $K_R^+ = 3.8 \times 10^{-10}$ mol^{-1} m^3 s^{-1} is is larger than 10.

$\rho_p = \rho_r$	K_R^+	Q_1
0.0005	4.2×10^{-7}	1.00059
0.0009	4.2×10^{-7}	12.3813
0.001	4.2×10^{-7}	785.766
0.005	4.2×10^{-7}	785.773
0.01	4.2×10^{-7}	785.762
0.02	4.2×10^{-7}	785.767
0.05	4.2×10^{-7}	785.770
0.1	4.2×10^{-7}	785.769
0.001	4×10^{-8}	1.00527
0.006	4×10^{-8}	8.43948
0.007	4×10^{-8}	785.770
0.01	4×10^{-8}	785.770
0.02	4×10^{-8}	785.767
0.05	4×10^{-8}	785.769

$\rho_p = \rho_r$	K_R^+	Q_1
0.1	4×10^{-8}	785.738
0.001	4×10^{-9}	1
0.005	4×10^{-9}	1.00045
0.01	4×10^{-9}	1.0527
0.02	4×10^{-9}	7.32281
0.045	4×10^{-9}	16.1847
0.05	4×10^{-9}	785.657
0.1	4×10^{-9}	784.726
0.001	3.8×10^{-10}	1
0.005	3.8×10^{-10}	1
0.01	3.8×10^{-10}	1
0.02	3.8×10^{-10}	1.00001
0.05	3.8×10^{-10}	1.0033
0.1	3.8×10^{-10}	1.39119

Variation of K_R^-

The depolymerisation rate of polynucleotides must be small enough before polymerases can be selectively generated. We found that the critical concentration increased from 0.0009 mol m^{-3} for $K_R^- = 8 \times 10^{-9}$ s^{-1} to 0.01 mol m^{-3} for $K_R^- = 1 \times 10^{-6}$ s^{-1}.

$\rho_p = \rho_r$	K_R^-	Q_1
0.0005	1×10^{-6}	1
0.001	1×10^{-6}	1
0.002	1×10^{-6}	1
0.005	1×10^{-6}	1
0.01	1×10^{-6}	1.00019
0.05	1×10^{-6}	9.8478
0.1	1×10^{-6}	416.687
0.2	1×10^{-6}	785.764
0.0005	1×10^{-7}	1
0.001	1×10^{-7}	1.00002
0.002	1×10^{-7}	1.00213
0.005	1×10^{-7}	1.8933
0.01	1×10^{-7}	785.771

$\rho_p = \rho_r$	K_R^-	Q_1
0.02	1×10^{-7}	785.743
0.05	1×10^{-7}	785.725
0.1	1×10^{-7}	783.479
0.0005	8×10^{-9}	1.29516
0.0009	8×10^{-9}	12.3813
0.001	8×10^{-9}	785.772
0.002	8×10^{-9}	785.764
0.005	8×10^{-9}	785.764
0.01	8×10^{-9}	785.770
0.02	8×10^{-9}	785.769
0.05	8×10^{-9}	785.768
0.1	8×10^{-9}	785.774

Variation of K_R^- and K_R^+ with K_R^+/K_R^- fixed

To confirm that it is the net polymerisation rate of polynucleotide that matters, we have varied K_R^- and K_R^+ simultaneously while keeping the ratio K_R^+/K_R^- constant and we observed that the critical concentration $\rho_p = \rho_r$ did not change as we varied these 2 parameters.

ρ	K_R^+	K_R^-	Q_1
0.0001	4.2×10^{-7}	8×10^{-9}	1.00001
0.0009	4.2×10^{-7}	8×10^{-9}	12.3813
0.001	4.2×10^{-7}	8×10^{-9}	785.773
0.002	4.2×10^{-7}	8×10^{-9}	785.773
0.005	4.2×10^{-7}	8×10^{-9}	785.773
0.01	4.2×10^{-7}	8×10^{-9}	785.772
0.02	4.2×10^{-7}	8×10^{-9}	785.773
0.05	4.2×10^{-7}	8×10^{-9}	785.772
0.1	4.2×10^{-7}	8×10^{-9}	785.773
0.0001	4.2×10^{-6}	8×10^{-8}	1.00001
0.0009	4.2×10^{-6}	8×10^{-8}	11.5881
0.001	4.2×10^{-6}	8×10^{-8}	20.6581
0.002	4.2×10^{-6}	8×10^{-8}	785.767
0.005	4.2×10^{-6}	8×10^{-8}	785.771

ρ	K_R^+	K_R^-	Q_1
0.01	4.2×10^{-6}	8×10^{-8}	785.772
0.02	4.2×10^{-6}	8×10^{-8}	785.773
0.05	4.2×10^{-6}	8×10^{-8}	785.769
0.1	4.2×10^{-6}	8×10^{-8}	785.794
0.0001	4.2×10^{-5}	8×10^{-7}	1.00001
0.0009	4.2×10^{-5}	8×10^{-7}	11.5251
0.001	4.2×10^{-5}	8×10^{-7}	20.3005
0.002	4.2×10^{-5}	8×10^{-7}	519.732
0.005	4.2×10^{-5}	8×10^{-7}	785.793
0.01	4.2×10^{-5}	8×10^{-7}	785.815
0.02	4.2×10^{-5}	8×10^{-7}	786.012
0.05	4.2×10^{-5}	8×10^{-7}	786.042
0.1	4.2×10^{-5}	8×10^{-7}	784.750

Variation of k_{step}

Increasing k_{step} to $0.02\,\mathrm{s}^{-1}$ increased the critical concentration by a factor of 2 but the value of Q_1 remained the same.

$\rho_p = \rho_r$	k_{step}	Q_1
0.0001	4×10^{-5}	1.00001
0.0002	4×10^{-5}	1.00076
0.0005	4×10^{-5}	1.29516
0.0009	4×10^{-5}	12.3813
0.001	4×10^{-5}	785.772
0.002	4×10^{-5}	785.764
0.005	4×10^{-5}	785.764
0.01	4×10^{-5}	785.770
0.02	4×10^{-5}	785.769
0.05	4×10^{-5}	785.768
0.1	4×10^{-5}	785.774

$\rho_p = \rho_r$	k_{step}	Q_1
0.0001	0.02	1
0.0002	0.02	1
0.0005	0.02	1.00059
0.001	0.02	1.0399
0.0016	0.02	1.69504
0.0017	0.02	785.745
0.002	0.02	785.776
0.005	0.02	785.770
0.01	0.02	785.768
0.02	0.02	785.757
0.05	0.02	785.799
0.1	0.02	785.747

Variation of Z

Increasing Z a hundred fold to $10^8\,\mathrm{mol^{-1}\,m^3\,s^{-1}}$ decreased the critical concentration by a factor of 3 to $0.0005\,\mathrm{mol\,m^{-3}}$ while increasing Z to $10^4\,\mathrm{mol^{-1}\,m^3\,s^{-1}}$ increased the critical concentration to $0.003\,\mathrm{mol\,m^{-3}}$.

ρ	Z	Q_1
0.0009	1×10^6	12.3813
0.001	1×10^6	785.774
0.005	1×10^6	785.775
0.01	1×10^6	785.762
0.02	1×10^6	785.774
0.05	1×10^6	785.761
0.1	1×10^6	785.774

ρ	Z	Q_1
0.0004	1×10^8	8.6133
0.0005	1×10^8	785.765
0.001	1×10^8	785.766
0.005	1×10^8	785.773
0.01	1×10^8	785.762
0.02	1×10^8	785.767
0.05	1×10^8	785.770
0.1	1×10^8	785.769

ρ	Z	Q_1
0.001	1×10^4	1.19743
0.002	1×10^4	10.0781
0.003	1×10^4	785.715
0.004	1×10^4	785.722
0.005	1×10^4	785.749
0.01	1×10^4	785.741
0.02	1×10^4	785.740
0.05	1×10^4	785.740

Variation of h_R

The rate of attachment of a free polynucleotide to a polynucleotide, h_R, has no significant effect on the critical concentration nor the value of Q_1 even for rates as low as $1\,\mathrm{mol^{-1}\,m^3\,s^{-1}}$.

$\rho_p = \rho_r$	h_R+	Q_1
0.0001	1	1.00001
0.0002	1	1.00092
0.0005	1	1.32139
0.001	1	785.506
0.002	1	785.564
0.005	1	785.717
0.01	1	785.507
0.02	1	785.504
0.05	1	785.735
0.1	1	785.635
0.0001	100	1.00001
0.0002	100	1.00076
0.0005	100	1.29542
0.001	100	785.595

$\rho_p = \rho_r$	h_R	Q_1
0.002	100	785.638
0.005	100	785.486
0.01	100	785.676
0.02	100	785.601
0.05	100	784.819
0.1	100	785.687
0.0001	10000	1.00001
0.0002	10000	1.00076
0.0005	10000	1.29516
0.001	10000	784.923
0.002	10000	785.245
0.005	10000	785.440
0.01	10000	785.659

$\rho_p = \rho_r$	h_R	Q_1
0.02	10000	785.658
0.05	10000	785.668
0.1	10000	785.160
0.0001	10^6	1.00001
0.0002	10^6	1.00076
0.0005	10^6	1.29516
0.001	10^6	785.772
0.002	10^6	785.764
0.005	10^6	785.764
0.01	10^6	785.770
0.02	10^6	785.769
0.05	10^6	785.768
0.1	10^6	785.774

Variation of $k^+_{P,1}$

The peptide polymerisation rate on a polynucleotide chain also has a very small effect. When varying $k^+_{P,1}$ in the range $1\times10^{-8}\,\mathrm{mol^{-1}\,m^3\,s^{-1}}$ to $0.1\,\mathrm{mol^{-1}\,m^3\,s^{-1}}$, we found that the critical concentrations only changed by a factor of 2.

$\rho_p = \rho_r$	$k^+_{P,1}$	Q_1
0.0001	1×10^{-8}	1.00001
0.0002	1×10^{-8}	1.00076
0.0005	1×10^{-8}	1.29506
0.001	1×10^{-8}	20.3446
0.002	1×10^{-8}	785.774
0.005	1×10^{-8}	785.774
0.01	1×10^{-8}	785.775
0.02	1×10^{-8}	785.777
0.05	1×10^{-8}	785.781
0.1	1×10^{-8}	785.790
0.0001	0.0001	1.00001
0.0002	0.0001	1.00076
0.0005	0.0001	1.29515

$\rho_p = \rho_r$	$k^+_{P,1}$	Q_1
0.001	0.0001	785.778
0.005	0.0001	785.775
0.01	0.0001	785.776
0.02	0.0001	785.776
0.1	0.0001	785.775
0.0001	0.05	1.00001
0.0002	0.05	1.00076
0.0005	0.05	1.29516
0.001	0.05	785.774
0.002	0.05	785.775
0.005	0.05	785.774
0.01	0.05	785.774

$\rho_p = \rho_r$	$k^+_{P,1}$	Q_1
0.02	0.05	785.773
0.05	0.05	785.773
0.1	0.05	785.773
0.0005	0.1	1.29516
0.0009	0.1	12.3813
0.001	0.1	785.772
0.002	0.1	785.764
0.005	0.1	785.764
0.01	0.1	785.770
0.02	0.1	785.769
0.05	0.1	785.768
0.1	0.1	785.774

Variation of λ

We also used 2 other values of λ and found that the critical concentration did not change but the value of Q_1 increased as λ decreased.

ρ	λ	Q_1
0.0009	0.1	12.5722
0.001	0.1	7565.74
0.005	0.1	7548.68
0.01	0.1	5867.70
0.02	0.1	7517.69
0.05	0.1	7898.22
0.1	0.1	7546.92

ρ	λ	Q_1
0.0009	0.15	12.3813
0.001	0.15	785.772
0.005	0.15	785.773
0.01	0.15	785.770
0.02	0.15	785.769
0.05	0.15	785.770
0.1	0.15	785.774

ρ	λ	Q_1
0.0009	0.2	11.6033
0.001	0.2	148.416
0.005	0.2	148.416
0.01	0.2	148.415
0.02	0.2	148.415
0.05	0.2	148.415
0.1	0.2	148.415

References

Bernhardt HS. Tate WP. 2012. Primordial soup or vinaigrette: did the RNA world evolve at acidic pH? Biol. Direct 7:4.

Florian J. Goodman MF. Warshel A. 2003. Computer simulation of the chemical catalysis of DNA polymerases: discriminating between alternative nucleotide insertion mechanisms for T7 DNA polymerase. J. Am. Chem. Soc. 125:8163-8177.

Hyeon C, Dima RI, Thirumalai D. 2006. Size, shape, and flexibility of RNA structures. J. Chem. Phys. 125:194905.

Kahne D, Still WC. 1988. Hydrolysis of a peptide bond in neutral water. J. Am. Chem. Soc. 110:7529-7534.

Lawrence MS, Bartel DP. 2003. Processivity of ribozyme-catalyzed RNA polymerization. Biochemistry 42:8748-55.

Radzicka A, Wolfenden R. 1996. Rates of uncatalyzed peptide bond hydrolysis in neutral solution and the transition state affinities of proteases. J. Am. Chem. Soc. 118:6105-6109.

Sievers A, Beringer M, Rodnina MV, Wolfenden R. 2004. The ribosome as an entropy trap. Proc. Natl. Acad. Sci. USA 101:7897-7901.

Wohlgemuth I, Beringer M, Rodnina MV. (2006), Rapid peptide bond formation on isolated 50S ribosomal subunits. EMBO Rep. 7:699-703.

Wolfenden R. 2014. Primordial chemistry and enzyme evolution in a hot environment. Cell. Mol. Life Sci. 71:2909-2915.

Wolfenden R, Snider MJ. 2001. The depth of chemical time and the power of enzymes as catalysts. Acc. Chem. Res. 34:938-945.
